# Supplementary figures and images for: Refining the Martin–Hopkins method for estimating low-density lipoprotein cholesterol levels: Median versus optimal TG/VLDL-C ratio
Source: PLoS One. 2025 Jul 3;20(7):e0327169. doi: 10.1371/journal.pone.0327169 (PMC12225850; doi:10.1371/journal.pone.0327169)

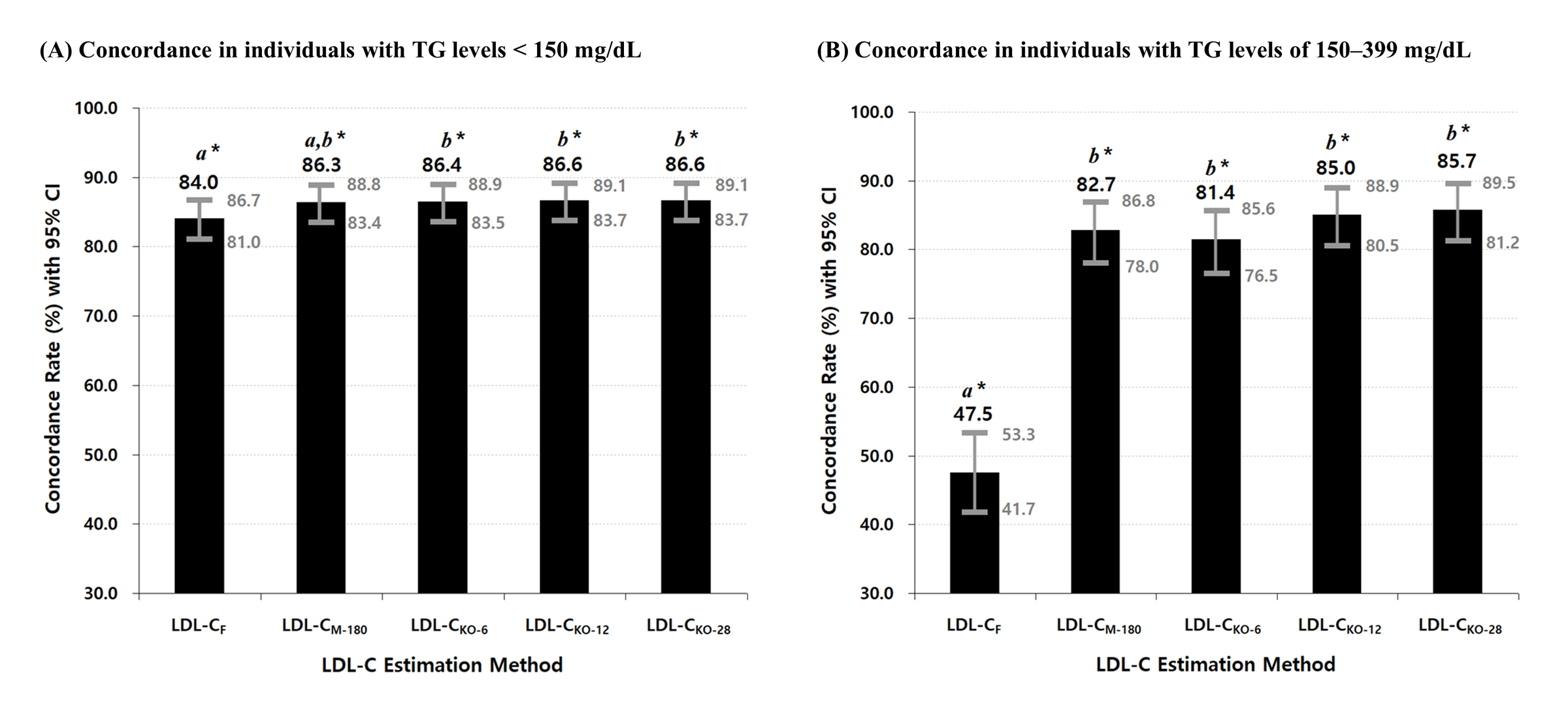

Supplement: S1 Fig — Bars represent concordance rates ± 95% confidence intervals for LDL-C estimates, stratified by triglyceride levels of < 150 mg/dL (Panel A) and 150–399 mg/dL (Panel B). From left to right, bars correspond to the following LDL-C estimates: LDL-CF, LDL-CM-180, LDL-CKO-6, LDL-CKO-12, and LDL-CKO-28. * Bars labeled with the same letters indicate no statistically significant difference in concordance between LDL-C estimates (α = 0.05), based on pairwise comparisons using McNemar’s exact test for correlated proportions (see S15 and S16 Tables for details). The 95% confidence intervals for concordance rates were calculated using the Clopper–Pearson exact method. Abbreviations: NCEP–ATP III: National Cholesterol Education Program Adult Treatment Panel III; TG: triglyceride; LDL-C: low-density lipoprotein cholesterol; LDL-CF: LDL-C calculated using the Friedewald formula; LDL-CM-180: LDL-C calculated using the original 180-cell Martin–Hopkins equation proposed by Martin et al. [14]; LDL-CKO-N (LDL-CKO-6-TG, LDL-CKO-12, and LDL-CKO-28): LDL-C calculated using the N-cell tables for the optimal TG/VLDL-C ratios derived from our dataset. (TIF) [file pone.0327169.s001.tif]
