# Supplementary material for: Refining the Martin–Hopkins method for estimating low-density lipoprotein cholesterol levels: Median versus optimal TG/VLDL-C ratio
Source: PLoS One. 2025 Jul 3;20(7):e0327169. doi: 10.1371/journal.pone.0327169 (PMC12225850; doi:10.1371/journal.pone.0327169)
Supplement: S1 Table — (DOCX) [file pone.0327169.s002.docx]

|  | Sample | | Population 1 | | Population 2 | | KNHSP 2015 | |
| --- | --- | --- | --- | --- | --- | --- | --- | --- |
| TGs, mg/dL | *n* | % | *n* | % | *n* | % | *n* | % |
| < 50 | 1,140 | 5.8 | 1,140 | 9.3 | 0 | 0.0 | 1,092,748 | 7.8 |
| 50–99 | 4,648 | 23.6 | 4,647 | 38.1 | 1 | 0.0 | 5,416,881 | 38.6 |
| 100–149 | 3,127 | 15.9 | 3,125 | 25.6 | 2 | 0.0 | 3,624,582 | 25.8 |
| 150–199 | 1,523 | 7.7 | 1,518 | 12.4 | 5 | 0.1 | 1,826,201 | 13.0 |
| 200–299 | 6,132 | 31.2 | 1,122 | 9.2 | 5,010 | 67.2 | 1,373,579 | 9.8 |
| 300–399 | 1,752 | 8.9 | 378 | 3.1 | 1,374 | 18.4 | 409,245 | 2.9 |
| ≥ 400 *^a^* | 1,342 | 6.8 | 275 | 2.3 | 1,067 | 14.3 | 281,059 | 2.0 |
| Total | 19,664 | 100.0 | 12,205 | 100.0 | 7,459 | 100.0 | 14,024,331 | 100.0 |

**Abbreviations:** KNHSP: Korea National Health Screening Program; TG: triglyceride.

*^a^* Participants with triglyceride levels ≥ 400 mg/dL were excluded from the study, as the Friedewald equation is not valid for such cases.
