# Supplementary material for: Refining the Martin–Hopkins method for estimating low-density lipoprotein cholesterol levels: Median versus optimal TG/VLDL-C ratio
Source: PLoS One. 2025 Jul 3;20(7):e0327169. doi: 10.1371/journal.pone.0327169 (PMC12225850; doi:10.1371/journal.pone.0327169)
Supplement: S2 Table — (DOCX) [file pone.0327169.s003.docx]

|  | | TG/VLDL-C ratio | |
| --- | --- | --- | --- |
| Triglycerides, mg/dL | *n* | Median (95% CI, ACL) *^a^* | Optimal *^b^* |
| < 40 | 462 | **2.6** (2.53–2.80, 95.5%) | **2.6** |
| 40–49 | 678 | **3.3** (3.21–3.45, 95.8%) | **3.6** |
| 50–74 | 2,385 | **4.1** (4.04–4.17, 95.1%) | **4.4** |
| 75–99 | 2,263 | **4.8** (4.70–4.86, 95.2%) | **4.8** |
| 100–124 | 1,813 | **5.3** (5.29–5.42, 95.2%) | **5.6** |
| 125–149 | 1,314 | **5.5** (5.43–5.63, 95.6%) | **6.2** |
| 150–174 | 871 | **5.8** (5.67–5.96, 95.1%) | **6.4** |
| 175–199 | 652 | **5.9** (5.78–6.01, 95.4%) | **5.9** |
| 200–249 | 4,056 | **6.2** (6.11–6.23, 95.0%) | **6.4** |
| 250–299 | 2,076 | **6.2** (6.17–6.33, 95.4%) | **6.1** |
| 300–349 | 1,102 | **6.4** (6.29–6.50, 95.6%) | **6.8** |
| 350–399 | 650 | **6.5** (6.39–6.63, 95.5%) | **6.5** |

**Abbreviations:** TG/VLDL-C ratio: ratio of triglycerides to very-low-density lipoprotein cholesterol; CI: confidence interval; ACL: actual confidence level.

*^a^* The 95% confidence interval for the median was constructed without assuming any specific distribution of the TG/VLDL-C ratio. The actual coverage may exceed 95%.

*^b^* The optimal TG/VLDL-C ratio was defined as the value that maximized concordance between estimated and directly measured LDL-C levels, according to the classification criteria of the National Cholesterol Education Program Adult Treatment Panel III (NCEP–ATP III) guideline.
