# Supplementary material for: Refining the Martin–Hopkins method for estimating low-density lipoprotein cholesterol levels: Median versus optimal TG/VLDL-C ratio
Source: PLoS One. 2025 Jul 3;20(7):e0327169. doi: 10.1371/journal.pone.0327169 (PMC12225850; doi:10.1371/journal.pone.0327169)
Supplement: S3 Table — (DOCX) [file pone.0327169.s004.docx]

|  | TG/VLDL-C ratio | | | | | |
| --- | --- | --- | --- | --- | --- | --- |
|  | Non–HDL-C < 130 mg/dL | | | Non–HDL-C ≥ 130 mg/dL | | |
| TGs, mg/dL | *n* | Median (95% CI, ACL) *^a^* | Optimal *^b^* | *n* | Median (95% CI, ACL) *^a^* | Optimal *^b^* |
| < 100 | 3,894 | **4.3** (4.21–4.34, 95.1%) | **4.8** | 1,894 | **4.1** (4.02–4.18. 95.4%) | **4.1** |
| 100–149 | 1,211 | **5.7** (5.63–5.84, 95.6%) | **6.1** | 1,916 | **5.2** (5.10–5.26, 95.3%) | **5.2** |
| 150–199 | 400 | **6.4** (6.26–6.60, 96.0%) | **6.4** | 1,123 | **5.7** (5.58–5.78, 95.1%) | **5.9** |
| 200–399 | 1,438 | **7.0** (6.91–7.09, 95.2%) | **6.8** | 6,446 | **6.1** (6.05–6.13, 95.2%) | **6.4** |
| ≥ 400 | 111 | **8.0** (7.58–8.33, 96.4%) | **7.3** | 1,231 | **6.8** (6.70–6.96, 95.4%) | **6.8** |

**Abbreviations:** TG/VLDL-C ratio: ratio of triglycerides to very-low-density lipoprotein cholesterol; TG: triglyceride; CI: confidence interval; ACL: actual confidence level.

*^a^* The 95% confidence interval for the median was constructed without assuming any specific distribution of the TG/VLDL-C ratio. The actual coverage may exceed 95%.

*^b^* The optimal TG/VLDL-C ratio was defined as the value that maximized concordance between estimated and directly measured LDL-C levels, according to the National Cholesterol Education Program Adult Treatment Panel III (NCEP–ATP III) guideline classification.
