# Supplementary material for: Refining the Martin–Hopkins method for estimating low-density lipoprotein cholesterol levels: Median versus optimal TG/VLDL-C ratio
Source: PLoS One. 2025 Jul 3;20(7):e0327169. doi: 10.1371/journal.pone.0327169 (PMC12225850; doi:10.1371/journal.pone.0327169)
Supplement: S4 Table — (DOCX) [file pone.0327169.s005.docx]

|  | Non–HDL-C, mg/dL *^a^* | | | | | |
| --- | --- | --- | --- | --- | --- | --- |
| Triglycerides, mg/dL | < 100 | 100–129 | 130–159 | 160–189 | 190–219 | ≥ 220 |
| < 49 | 3.3 (516) | 3.0 (433) | 2.7 (145) | 2.8 (37) | 1.8 (5) | 2.1 (4) |
| 50–56 | 4.1 (218) | 3.7 (278) | 3.5 (124) | 3.2 (27) | 8.1 (2) |  |
| 57–61 | 4.3 (151) | 4.2 (199) | 3.9 (109) | 3.2 (17) | 4.9 (2) | 2.6 (2) |
| 62–66 | 4.5 (121) | 4.1 (205) | 3.9 (121) | 3.6 (25) | 2.9 (4) |  |
| 67–71 | 4.6 (122) | 4.4 (209) | 3.9 (111) | 3.8 (38) | 3.2 (3) |  |
| 72–75 | 4.8 (84) | 4.6 (142) | 4.5 (109) | 4.3 (32) | 4.4 (10) |  |
| 76–79 | 4.7 (83) | 4.7 (167) | 4.5 (116) | 3.5 (36) | 5.0 (7) |  |
| 80–83 | 5.0 (75) | 4.7 (146) | 4.5 (113) | 4.3 (41) | 4.1 (9) | 5.2 (1) |
| 84–87 | 4.4 (51) | 4.8 (146) | 4.7 (103) | 4.6 (28) | 4.4 (17) | 4.1 (2) |
| 88–92 | 5.5 (78) | 5.1 (166) | 4.9 (126) | 4.3 (67) | 3.9 (12) | 2.6 (3) |
| 93–96 | 5.3 (60) | 5.1 (149) | 4.6 (102) | 4.2 (51) | 4.0 (7) | 5.7 (4) |
| 97–100 | 5.8 (39) | 5.3 (88) | 4.8 (100) | 4.8 (43) | 5.2 (15) | 2.6 (1) |
| 101–105 | 5.7 (56) | 5.6 (118) | 5.0 (157) | 4.6 (55) | 4.5 (22) | 3.4 (2) |
| 106–110 | 5.7 (52) | 5.5 (123) | 5.1 (135) | 4.6 (49) | 4.5 (24) | 4.6 (4) |
| 111–115 | 5.8 (35) | 5.7 (123) | 5.6 (106) | 4.9 (64) | 5.0 (22) | 3.0 (1) |
| 116–120 | 5.9 (36) | 5.7 (105) | 5.3 (105) | 5.3 (72) | 4.9 (23) | 3.5 (6) |
| 121–126 | 5.9 (39) | 5.6 (95) | 5.6 (139) | 5.0 (72) | 4.5 (18) | 4.3 (7) |
| 127–132 | 5.6 (32) | 5.6 (80) | 5.7 (128) | 5.3 (75) | 4.5 (28) | 4.4 (9) |
| 133–138 | 6.3 (31) | 6.0 (81) | 5.6 (105) | 5.2 (55) | 4.7 (28) | 4.2 (6) |
| 139–146 | 5.5 (26) | 6.1 (106) | 5.7 (135) | 5.3 (91) | 4.9 (29) | 3.5 (5) |
| 147–154 | 6.8 (19) | 6.0 (73) | 5.7 (112) | 5.4 (86) | 5.7 (26) | 4.7 (13) |
| 155–163 | 5.9 (16) | 6.5 (84) | 5.8 (101) | 5.4 (75) | 4.8 (24) | 4.2 (14) |
| 164–173 | 6.5 (18) | 6.6 (77) | 5.9 (122) | 5.9 (90) | 4.4 (29) | 5.1 (6) |
| 174–185 | 6.8 (18) | 5.9 (70) | 6.0 (121) | 5.9 (96) | 5.4 (37) | 5.0 (5) |
| 186–201 | 6.8 (29) | 6.7 (84) | 5.9 (196) | 5.9 (144) | 5.3 (68) | 4.7 (29) |
| 202–220 | 7.3 (89) | 6.8 (323) | 6.3 (614) | 6.0 (497) | 5.4 (254) | 5.1 (92) |
| 221–247 | 7.0 (65) | 6.8 (320) | 6.4 (570) | 6.0 (540) | 5.8 (269) | 5.4 (102) |
| 248–292 | 7.7 (55) | 7.1 (279) | 6.4 (590) | 6.0 (570) | 5.7 (323) | 5.1 (125) |
| 293–399 | 7.9 (47) | 7.1 (213) | 6.6 (575) | 6.3 (645) | 5.9 (341) | 5.6 (170) |
| ≥ 400 | 7.7 (11) | 8.0 (100) | 7.3 (285) | 7.0 (418) | 6.7 (275) | 6.1 (253) |

**Abbreviations:** TG/VLDL-C ratio: ratio of triglycerides to very-low-density lipoprotein cholesterol; non–HDL-C: non–high-density lipoprotein cholesterol.

*^a^* The values in the table represent the median TG/VLDL-C ratios, with the number of subjects shown in parentheses.
