# Supplementary material for: Refining the Martin–Hopkins method for estimating low-density lipoprotein cholesterol levels: Median versus optimal TG/VLDL-C ratio
Source: PLoS One. 2025 Jul 3;20(7):e0327169. doi: 10.1371/journal.pone.0327169 (PMC12225850; doi:10.1371/journal.pone.0327169)
Supplement: S5 Table — (DOCX) [file pone.0327169.s006.docx]

|  | | | TG/VLDL-C ratio | |
| --- | --- | --- | --- | --- |
| Triglycerides, mg/dL | Non–HDL-C, mg/dL | *n* | Median (95% CI, ACL) *^a^* | Optimal *^b^* |
| < 50 | < 100 | 516 | **3.3** (3.18–3.41, 95.3%) | **4.2** |
|  | 100–129 | 433 | **3.0** (2.90–3.20, 95.7%) | **3.9** |
|  | ≥ 130 | 191 | **2.7** (2.58–2.89, 95.8%) | **2.5** |
| 50–99 | < 100 | 1,071 | **4.6** (4.23–4.75, 95.6%) | **4.8** |
|  | 100–129 | 1,874 | **4.5** (4.43–4.61, 95.0%) | **4.8** |
|  | 130–159 | 1,210 | **4.3** (4.18–4.40, 95.3%) | **4.1** |
|  | ≥ 160 | 493 | **4.0** (3.91–4.19, 95.3%) | **3.9** |
| 100–149 | < 100 | 326 | **5.8** (5.57–6.01, 96.0%) | **6.1** |
|  | 100–129 | 885 | **5.7** (5.60–5.83, 95.6%) | **6.1** |
|  | 130–159 | 1,090 | **5.4** (5.29–5.55, 95.1%) | **5.6** |
|  | 160–189 | 576 | **5.0** (4.95–5.22, 95.9%) | **5.2** |
|  | ≥ 190 | 250 | **4.6** (4.42–4.83, 95.0%) | **4.8** |
| 150–199 | < 100 | 174 | **6.7** (6.21–7.18, 95.3%) | **7.6** |
|  | 100–129 | 226 | **6.4** (6.14–6.56, 96.0%) | **6.4** |
|  | 130–159 | 525 | **5.9** (5.75–6.03, 95.5%) | **5.9** |
|  | 160–189 | 403 | **5.7** (5.53–5.87, 95.4%) | **5.8** |
|  | ≥ 190 | 195 | **4.8** (4.67–5.24, 95.5%) | **4.6** |
| 200–299 | < 100 | 232 | **7.3** (7.03–7.45, 95.8%) | **7.7** |
|  | 100–129 | 985 | **6.9** (6.73–6.47, 95.2%) | **6.8** |
|  | 130–159 | 1922 | **6.4** (6.28–6.47, 95.3%) | **6.6** |
|  | 160–189 | 1729 | **6.0** (5.95–6.08, 95.1%) | **6.1** |
|  | 190–219 | 910 | **5.7** (5.54–5.79, 95.7%) | **5.9** |
|  | ≥ 220 | 354 | **5.2** (5.06–5.46, 95.1%) | **6.0** |
| 300–399 | < 130 | 221 | **7.3** (7.09–7.44, 95.7%) | **7.7** |
|  | 130–159 | 498 | **6.7** (6.52–6.81, 95.6%) | **6.9** |
|  | 160–189 | 579 | **6.3** (6.15–6.40, 95.4%) | **6.4** |
|  | 190–219 | 300 | **5.9** (5.72–6.21, 95.7%) | **6.0** |
|  | ≥ 220 | 154 | **5.6** (5.28–5.78, 95.6%) | **5.7** |

**Abbreviations:** TG/VLDL-C ratio: ratio of triglycerides to very-low-density lipoprotein cholesterol; non–HDL-C: non–high-density lipoprotein cholesterol; CI: confidence interval; ACL: actual confidence level.

*^a^* The 95% confidence interval for the median was constructed without assuming any specific distribution of the TG/VLDL-C ratio. The actual coverage may exceed 95%.

*^b^* The optimal TG/VLDL-C ratio was defined as the value that maximized concordance between estimated and directly measured LDL-C, according to the National Cholesterol Education Program Adult Treatment Panel III (NCEP–ATP III) guideline classification.
