# Supplementary material for: Refining the Martin–Hopkins method for estimating low-density lipoprotein cholesterol levels: Median versus optimal TG/VLDL-C ratio
Source: PLoS One. 2025 Jul 3;20(7):e0327169. doi: 10.1371/journal.pone.0327169 (PMC12225850; doi:10.1371/journal.pone.0327169)
Supplement: S6 Table — (DOCX) [file pone.0327169.s007.docx]

|  | Estimated LDL-C (LDL-C_E_) *^a^* | | | | | | | | | | | | |
| --- | --- | --- | --- | --- | --- | --- | --- | --- | --- | --- | --- | --- | --- |
| LDL-C_E_ | M-10 | M-180 | KM-6-TG | KO-6-TG | KM-10 | KO-10 | KM-12-TG | KO-12-TG | KM-12 | KO-12 | KM-28 | KO-28 | KM-180 |
| LDL-C_F_ | < 0.001 | < 0.001 | < 0.001 | < 0.001 | < 0.001 | < 0.001 | < 0.001 | < 0.001 | < 0.001 | < 0.001 | < 0.001 | < 0.001 | < 0.001 |
| LDL-C_M-10_ | NA | 0.045 | 0.308 | 0.027 | 1.000 | 0.008 | 0.089 | 0.002 | 0.008 | < 0.001 | 0.001 | < 0.001 | < 0.001 |
| LDL-C_M-180_ | 0.045 | NA | 0.298 | 1.000 | 0.063 | 0.969 | 0.792 | 0.253 | 0.967 | 0.032 | 0.305 | < 0.001 | 0.003 |
| LDL-C_KM-6-TG_ *^b^* | 0.308 | 0.298 | NA | 0.089 | 0.323 | 0.271 | 0.226 | 0.011 | 0.162 | 0.001 | 0.016 | < 0.001 | < 0.001 |
| LDL-C_KO-6-TG_ *^b^* | 0.027 | 1.000 | 0.089 | NA | 0.053 | 0.933 | 0.768 | 0.135 | 0.917 | 0.013 | 0.243 | < 0.001 | 0.009 |
| LDL-C_KM-10_ | 1.000 | 0.063 | 0.323 | 0.053 | NA | 0.013 | 0.095 | 0.004 | 0.006 | < 0.001 | 0.001 | < 0.001 | < 0.001 |
| LDL-C_KO-10_ | 0.008 | 0.969 | 0.271 | 0.933 | 0.013 | NA | 0.761 | 0.286 | 1.000 | 0.003 | 0.365 | < 0.001 | 0.033 |
| LDL-C_KM-12-TG_ *^b^* | 0.089 | 0.792 | 0.226 | 0.768 | 0.095 | 0.761 | NA | 0.087 | 0.727 | 0.014 | 0.204 | < 0.001 | 0.004 |
| LDL-C_KO-12-TG_ *^b^* | 0.002 | 0.253 | 0.011 | 0.135 | 0.004 | 0.286 | 0.087 | NA | 0.284 | 0.324 | 0.907 | 0.007 | 0.222 |
| LDL-C_KM-12_ | 0.008 | 0.967 | 0.162 | 0.917 | 0.006 | 1.000 | 0.727 | 0.284 | NA | 0.006 | 0.183 | < 0.001 | 0.012 |
| LDL-C_KO-12_ | < 0.001 | 0.032 | 0.001 | 0.013 | < 0.001 | 0.003 | 0.014 | 0.324 | 0.006 | NA | 0.254 | 0.020 | 0.745 |
| LDL-C_KM-28_ | < 0.001 | 0.305 | 0.016 | 0.243 | < 0.001 | 0.365 | 0.204 | 0.907 | 0.183 | 0.254 | NA | < 0.001 | 0.091 |
| LDL-C_KO-28_ | < 0.001 | < 0.001 | < 0.001 | < 0.001 | < 0.001 | < 0.001 | < 0.001 | 0.007 | < 0.001 | 0.020 | < 0.001 | NA | 0.174 |
| LDL-C_KM-180_ | < 0.001 | 0.003 | < 0.001 | 0.009 | < 0.001 | 0.033 | 0.004 | 0.222 | 0.012 | 0.745 | 0.091 | 0.174 | NA |

**Abbreviations:** LDL-C: low-density lipoprotein cholesterol; LDL-C_E_: estimated LDL-C; LDL-C_F_: LDL-C calculated using the Friedewald formula; LDL-C_M-N_ (LDL-C_M-10_ and LDL-C_M-180_): LDL-C calculated using the N-cell tables with the median ratios of triglycerides to very-low-density lipoprotein cholesterol (TG/VLDL-C) reported by Martin et al. [14]; LDL-C_KM-N_ (LDL-C_KM-6-TG_, LDL-C_KM-10_, LDL-C_KM-12-TG_, LDL-C_KM-12_, LDL-C_KM-28_, and LDL-C_KM-180_): LDL-C calculated using the N-cell tables with the median TG/VLDL-C ratios derived from our dataset; LDL-C_KO-N_ (LDL-C_KO-6-TG_, LDL-C_KO-10_, LDL-C_KO-12-TG_, LDL-C_KO-12_, and LDL-C_KO-28_): LDL-C calculated using the N-cell tables with the optimal TG/VLDL-C ratios derived from our dataset; NA: not applicable.

*^a^* The values in the table are *p*-values. Statistical significance of differences in overall concordance between two LDL-C estimates was assessed using McNemar’s exact test for correlated proportions.

*^b^* When stratification was based on TG levels alone rather than on combined TG and non–HDL-C levels, the subscript “_TG_” was added, as in LDL-C_KM-N-TG_ or LDL-C_KO-N-TG_.
