# Supplementary material for: Refining the Martin–Hopkins method for estimating low-density lipoprotein cholesterol levels: Median versus optimal TG/VLDL-C ratio
Source: PLoS One. 2025 Jul 3;20(7):e0327169. doi: 10.1371/journal.pone.0327169 (PMC12225850; doi:10.1371/journal.pone.0327169)
Supplement: S7 Table — (DOCX) [file pone.0327169.s008.docx]

|  | Triglyceride category, mg/dL | | | | | | |  |
| --- | --- | --- | --- | --- | --- | --- | --- | --- |
|  | < 50 | 50–99 | 100–149 | 150–199 | 200–299 | 300–399 | Overall |  |
| LDL-C_E_ | CN (%) | CN (%) | CN (%) | CN (%) | CN (%) | CN (%) | CN (%) | *p*-value *^a^* |
| Martin–Hopkins [14] | 978 (85.8) | 3,957 (85.2) | 2,635 (84.3) | 1,225 (80.7) | 866 (77.2) | 264 (69.8) | 9,925 (83.2) | NA |
| Sampson [15] | 937 (82.2) | 3,883 (83.6) | 2,623 (83.9) | 1,217 (80.2) | 877 (78.2) | 265 (70.1) | 9,802 (82.2) | < 0.001 |
| Rao [16] | 939 (82.4) | 3,920 (84.4) | 2,608 (83.5) | 1,198 (78.9) | 847 (75.5) | 262 (69.3) | 9,774 (81.9) | < 0.001 |
| Puavilai [17] | 940 (82.5) | 3,934 (84.7) | 2,550 (81.6) | 1,159 (76.4) | 866 (77.2) | 262 (69.3) | 9,711 (81.4) | < 0.001 |
| Chen [18] | 969 (85.0) | 3,858 (83.0) | 2,518 (80.6) | 1,211 (79.8) | 871 (77.6) | 279 (73.8) | 9,709 (81.4) | < 0.001 |
| DeLong [19] | 908 (79.6) | 3,794 (81.6) | 2,596 (83.1) | 1,215 (80.0) | 866 (77.2) | 269 (69.3) | 9,641 (80.8) | < 0.001 |
| Friedewald [6] | 940 (82.5) | 3,934 (84.7) | 2,550 (81.6) | 1,159 (76.4) | 734 (65.4) | 183 (48.4) | 9,500 (79.6) | < 0.001 |
| Vujovic [20] | 875 (76.8) | 3,652 (78.6) | 2,502 (80.1) | 1,187 (78.2) | 829 (73.9) | 276 (73.0) | 9,321 (78.1) | < 0.001 |
| McNamara [21] | 970 (85.1) | 3,944 (84.9) | 2,432 (77.8) | 1,029 (67.8) | 729 (65.0) | 183 (48.4) | 9,287 (77.8) | < 0.001 |
| Teerakanchana [22] | 819 (71.8) | 3,554 (76.5) | 2,527 (80.9) | 1,212 (79.8) | 854 (76.1) | 271 (71.7) | 9,237 (77.4) | < 0.001 |
| Dansethakul [23] | 667 (58.8) | 3,140 (67.6) | 2,411 (77.2) | 1,213 (79.9) | 864 (77.0) | 240 (63.5) | 8,535 (71.5) | < 0.001 |
| Hattori [24] | 974 (85.4) | 3,743 (80.5) | 2,164 (69.2) | 910 (59.9) | 590 (52.6) | 139 (36.8) | 8,520 (71.4) | < 0.001 |
| Saiedullah [25] | 810 (71.1) | 3,235 (69.6) | 2,112 (67.6) | 990 (65.2) | 680 (60.6) | 208 (55.0) | 8,035 (67.4) | < 0.001 |
| Rasouli [26] | 890 (78.1) | 3,174 (68.3) | 1,739 (55.6) | 790 (52.0) | 607 (54.1) | 204 (54.0) | 7,404 (62.1) | < 0.001 |
| Anandaraja [27] | 445 (39.0) | 2,670 (57.5) | 2,183 (69.9) | 1,040 (68.5) | 685 (61.1) | 171 (45.2) | 7,194 (60.3) | < 0.001 |
| DeCordova [28] | 629 (55.2) | 2,488 (53.5) | 1,769 (56.6) | 1,017 (67.0) | 802 (71.5) | 218 (57.7) | 6,923 (58.0) | < 0.001 |
| Ahmadi [29] | 307 (26.9) | 3,188 (68.6) | 969 (31.0) | 41 ( 2.7) | 27 ( 2.4) | 8 ( 2.1) | 4,540 (38.1) | < 0.001 |

**Abbreviations:** NCEP–ATP III: National Cholesterol Education Program Adult Treatment Panel III; LDL-C: low-density lipoprotein cholesterol; LDL-C_E_: estimated LDL-C; CN: concordant number; NA: not applicable.

*^a^* Statistical significance of differences in overall concordance between the Martin–Hopkins LDL-C estimates and other LDL-C estimates was assessed using McNemar’s exact test for correlated proportions.

LDL-C _Martin–Hopkins_: LDL-C calculated using the original 180-cell Martin–Hopkins equation.
