# Supplementary material for: Refining the Martin–Hopkins method for estimating low-density lipoprotein cholesterol levels: Median versus optimal TG/VLDL-C ratio
Source: PLoS One. 2025 Jul 3;20(7):e0327169. doi: 10.1371/journal.pone.0327169 (PMC12225850; doi:10.1371/journal.pone.0327169)
Supplement: S8 Table — (DOCX) [file pone.0327169.s009.docx]

|  | Model Fit | Error *^a^*, mg/dL | Absolute Error, mg/dL | Squared Error, mg/dL^2^ | Relative Error *^b^* , % |
| --- | --- | --- | --- | --- | --- |
| LDL-C_E_ | *R*-square | Mean (SD) | Mean (SD) | Mean (SD) | Mean (SD) |
| LDL-C_KO-28_ | 0.954 | 0.36 (6.89) | 5.09 (4.66) | 47.64 (236.93) | 0.73 (7.04) |
| Martin–Hopkins [14] | 0.952 | 0.98 (6.94) | 5.11 (4.80) | 49.15 (248.40) | 1.31 (7.21) |
| Sampson [15] | 0.948 | 1.54 (7.30) | 5.59 (4.95) | 55.70 (198.93) | 1.68 (7.31) |
| Rao [16] | 0.946 | 0.87 (7.47) | 5.63 (4.98) | 56.59 (247.93) | 1.06 (7.62) |
| Puavilai [17] | 0.943 | 0.35 (7.62) | 5.75 (5.02) | 58.20 (249.73) | 0.58 (7.74) |
| Chen [18] | 0.951 | −2.12 (7.04) | 5.61 (4.76) | 54.09 (210.74) | −1.24 (7.14) |
| DeLong [19] | 0.947 | 3.24 (7.37) | 6.09 (5.26) | 64.84 (254.78) | 3.32 (7.66) |
| Friedewald [6] | 0.932 | −0.76 (8.36) | 6.26 (5.59) | 70.39 (234.90) | −0.45 (8.57) |
| Vujovic [20] | 0.952 | 5.64 (7.09) | 6.99 (5.76) | 82.07 (283.71) | 5.59 (7.48) |
| McNamara [21] | 0.932 | −2.81 (8.36) | 6.67 (5.77) | 77.75 (236.94) | −2.43 (8.50) |
| Teerakanchana [22] | 0.937 | 4.92 (7.97) | 7.11 (6.09) | 87.65 (257.24) | 5.71 (9.61) |
| Dansethakul [23] | 0.931 | 6.88 (8.39) | 8.92 (6.19) | 117.83 (267.51) | 7.03 (9.29) |
| Hattori [24] | 0.932 | −7.02 (8.24) | 8.50 (6.70) | 117.21 (234.53) | −6.09 (8.09) |
| Saiedullah [25] | 0.952 | 9.92 (6.90) | 10.22 (6.45) | 145.96 (299.36) | 10.26 (9.07) |
| Rasouli [26] | 0.932 | −9.52 (10.17) | 11.37 (8.04) | 193.94 (267.03) | −6.95 (9.47) |
| Anandaraja [27] | 0.763 | 5.60 (15.69) | 12.78 (10.69) | 277.53 (504.85) | 6.97 (18.33) |
| DeCordova [28] | 0.894 | −10.21 (10.72) | 12.51 (7.93) | 219.28 (288.89) | −8.12 (10.08) |
| Ahmadi [29] | 0.398 | 23.26 (44.73) | 33.69 (37.50) | 2541.56 (5798.41) | 21.68 (47.01) |

**Abbreviations:** LDL-C: low-density lipoprotein cholesterol; LDL-C_KO-28_: LDL-C calculated using the 28-cell table (Fig 2) with the optimal ratios of triglycerides to very-low-density lipoprotein cholesterol (TG/VLDL-C) derived from our dataset; SD: standard deviation.

*^a^* Error = Estimated LDL-C (LDL-C_E_) – Directly measured LDL-C (LDL-C_D_).

*^b^* Relative error = [(LDL-C_E_ – LDL-C_D_) / LDL-C_D_] × 100

LDL-C _Martin–Hopkins_: LDL-C calculated using the original 180-cell Martin–Hopkins equation.
