# Supplementary material for: Refining the Martin–Hopkins method for estimating low-density lipoprotein cholesterol levels: Median versus optimal TG/VLDL-C ratio
Source: PLoS One. 2025 Jul 3;20(7):e0327169. doi: 10.1371/journal.pone.0327169 (PMC12225850; doi:10.1371/journal.pone.0327169)
Supplement: S9 Table — (DOCX) [file pone.0327169.s010.docx]

|  | Estimated LDL-C (LDL-C_E_) *^a^* | | | | | | | | | | | | |
| --- | --- | --- | --- | --- | --- | --- | --- | --- | --- | --- | --- | --- | --- |
| LDL-C_E_ | M-10 | M-180 | KM-6-TG | KO-6-TG | KM-10 | KO-10 | KM-12-TG | KO-12-TG | KM-12 | KO-12 | KM-28 | KO-28 | KM-180 |
| LDL-C_F_ | <0.001 | <0.001 | <0.001 | <0.001 | 0.001 | <0.001 | <0.001 | <0.001 | <0.001 | <0.001 | <0.001 | <0.001 | <0.001 |
| LDL-C_M-10_ | NA | 0.021 | 0.703 | 0.075 | 0.569 | 0.114 | 0.211 | 0.011 | 0.45 | <0.001 | 0.106 | <0.001 | 0.004 |
| LDL-C_M-180_ | 0.021 | NA | 0.044 | 0.465 | 0.01 | 0.354 | 0.244 | 0.7 | 0.635 | 0.296 | 0.342 | 0.048 | 0.211 |
| LDL-C_KM-6-TG_ *^b^* | 0.703 | 0.044 | NA | 0.069 | 0.39 | 0.444 | 0.226 | 0.012 | 0.072 | 0.004 | 0.192 | <0.001 | 0.004 |
| LDL-C_KO-6-TG_ *^b^* | 0.075 | 0.465 | 0.069 | NA | 0.069 | 0.787 | 0.757 | 0.193 | 0.835 | 0.063 | 0.794 | 0.008 | 0.085 |
| LDL-C_KM-10_ | 0.569 | 0.01 | 0.39 | 0.069 | NA | 0.04 | 0.103 | 0.006 | 0.008 | <0.001 | 0.047 | <0.001 | 0.001 |
| LDL-C_KO-10_ | 0.114 | 0.354 | 0.444 | 0.787 | 0.04 | NA | 1 | 0.212 | 0.568 | 0.003 | 0.961 | <0.001 | 0.094 |
| LDL-C_KM-12-TG_ *^b^* | 0.211 | 0.244 | 0.226 | 0.757 | 0.103 | 1 | NA | 0.121 | 0.646 | 0.065 | 0.006 | 0.01 | 0.038 |
| LDL-C_KO-12-TG_ *^b^* | 0.011 | 0.7 | 0.012 | 0.193 | 0.006 | 0.212 | 0.121 | NA | 0.401 | 0.534 | 0.208 | 0.126 | 0.62 |
| LDL-C_KM-12_ | 0.45 | 0.635 | 0.072 | 0.835 | 0.008 | 0.568 | 0.646 | 0.401 | NA | 0.062 | 0.498 | 0.004 | 0.142 |
| LDL-C_KO-12_ | <0.001 | 0.296 | 0.004 | 0.063 | <0.001 | 0.003 | 0.065 | 0.534 | 0.062 | NA | 0.039 | 0.142 | 0.961 |
| LDL-C_KM-28_ | 0.106 | 0.342 | 0.192 | 0.794 | 0.047 | 0.961 | 0.006 | 0.208 | 0.498 | 0.039 | NA | 0.001 | 0.041 |
| LDL-C_KO-28_ | <0.001 | 0.048 | <0.001 | 0.008 | <0.001 | <0.001 | 0.01 | 0.126 | 0.004 | 0.142 | 0.001 | NA | 0.001 |
| LDL-C_KM-180_ | 0.004 | 0.211 | 0.004 | 0.085 | 0.001 | 0.094 | 0.038 | 0.62 | 0.142 | 0.961 | 0.041 | 0.001 | NA |

**Abbreviations:** LDL-C: low-density lipoprotein cholesterol; LDL-C_E_: estimated LDL-C; LDL-C_F_: LDL-C calculated using the Friedewald formula; LDL-C_M-N_ (LDL-C_M-10_ and LDL-C_M-180_): LDL-C calculated using the N-cell tables with the median ratios of triglycerides to very-low-density lipoprotein cholesterol (TG/VLDL-C) reported by Martin et al. [14]; LDL-C_KM-N_ (LDL-C_KM-6-TG_, LDL-C_KM-10_, LDL-C_KM-12-TG_, LDL-C_KM-12_, LDL-C_KM-28_, and LDL-C_KM-180_): LDL-C calculated using the N-cell tables with the median TG/VLDL-C ratios derived from our dataset; LDL-C_KO-N_ (LDL-C_KO-6-TG_, LDL-C_KO-10_, LDL-C_KO-12-TG_, LDL-C_KO-12_, and LDL-C_KO-28_): LDL-C calculated using the N-cell tables with the optimal TG/VLDL-C ratios derived from our dataset; NA: not applicable.

*^a^* The values in the table are *p*-values. Statistical significance of differences in overall concordance between two LDL-C estimates was assessed using McNemar’s exact test for correlated proportions.

*^b^* When stratification was based on TG levels alone, rather than combined TG and non–HDL-C levels, the subscript “_TG_” was added, as in LDL-C_KM-N-TG_ or LDL-C_KO-N-TG_.
