# Supplementary material for: Refining the Martin–Hopkins method for estimating low-density lipoprotein cholesterol levels: Median versus optimal TG/VLDL-C ratio
Source: PLoS One. 2025 Jul 3;20(7):e0327169. doi: 10.1371/journal.pone.0327169 (PMC12225850; doi:10.1371/journal.pone.0327169)
Supplement: S10 Table — (DOCX) [file pone.0327169.s011.docx]

|  | Estimated LDL-C (LDL-C_E_) *^a^* | | | | | | | | | | | | |
| --- | --- | --- | --- | --- | --- | --- | --- | --- | --- | --- | --- | --- | --- |
| LDL-C_E_ | M-10 | M-180 | KM-6-TG | KO-6-TG | KM-10 | KO-10 | KM-12-TG | KO-12-TG | KM-12 | KO-12 | KM-28 | KO-28 | KM-180 |
| LDL-C_F_ | <0.001 | <0.001 | <0.001 | <0.001 | <0.001 | <0.001 | <0.001 | <0.001 | <0.001 | <0.001 | <0.001 | <0.001 | <0.001 |
| LDL-C_M-10_ | NA | 0.89 | 0.253 | 0.214 | 0.395 | 0.02 | 0.253 | 0.109 | 0.176 | 0.004 | 0.001 | <0.001 | 0.002 |
| LDL-C_M-180_ | 0.89 | NA | 0.449 | 0.443 | 0.687 | 0.128 | 0.449 | 0.223 | 0.45 | 0.034 | 0.002 | <0.001 | 0.003 |
| LDL-C_KM-6-TG_ *^b^* | 0.253 | 0.449 | NA | 1 | 0.681 | 0.353 | 1 | 0.512 | 1 | 0.066 | 0.041 | 0.008 | 0.048 |
| LDL-C_KO-6-TG_ *^b^* | 0.214 | 0.443 | 1 | NA | 1 | 0.275 | 1 | 0.516 | 1 | 0.095 | 0.044 | 0.008 | 0.049 |
| LDL-C_KM-10_ | 0.395 | 0.687 | 0.681 | 1 | NA | 0.198 | 0.681 | 0.341 | 0.542 | 0.008 | 0.004 | 0.001 | 0.012 |
| LDL-C_KO-10_ | 0.02 | 0.128 | 0.353 | 0.275 | 0.198 | NA | 0.353 | 0.92 | 0.379 | 0.416 | 0.13 | 0.026 | 0.195 |
| LDL-C_KM-12-TG_ *^b^* | 0.253 | 0.449 | 1 | 1 | 0.681 | 0.353 | NA | 0.512 | 1 | 0.066 | 0.028 | 0.008 | 0.048 |
| LDL-C_KO-12-TG_ *^b^* | 0.109 | 0.223 | 0.512 | 0.516 | 0.341 | 0.92 | 0.512 | NA | 0.55 | 0.419 | 0.155 | 0.039 | 0.194 |
| LDL-C_KM-12_ | 0.176 | 0.45 | 1 | 1 | 0.542 | 0.379 | 1 | 0.55 | NA | 0.025 | 0.007 | 0.003 | 0.026 |
| LDL-C_KO-12_ | 0.004 | 0.034 | 0.066 | 0.095 | 0.008 | 0.416 | 0.066 | 0.419 | 0.025 | NA | 0.344 | 0.081 | 0.455 |
| LDL-C_KM-28_ | 0.001 | 0.002 | 0.041 | 0.044 | 0.004 | 0.13 | 0.028 | 0.155 | 0.007 | 0.344 | NA | 0.363 | 0.92 |
| LDL-C_KO-28_ | <0.001 | <0.001 | 0.008 | 0.008 | 0.001 | 0.026 | 0.008 | 0.039 | 0.003 | 0.081 | 0.363 | NA | 0.363 |
| LDL-C_KM-180_ | 0.002 | 0.003 | 0.048 | 0.049 | 0.012 | 0.195 | 0.048 | 0.194 | 0.026 | 0.455 | 0.92 | 0.363 | NA |

**Abbreviations:** LDL-C: low-density lipoprotein cholesterol; LDL-C_E_: estimated LDL-C; LDL-C_F_: LDL-C calculated using the Friedewald formula; LDL-C_M-N_ (LDL-C_M-10_ and LDL-C_M-180_): LDL-C calculated using the N-cell tables with the median ratios of triglycerides to very-low-density lipoprotein cholesterol (TG/VLDL-C) reported by Martin et al. [14]; LDL-C_KM-N_ (LDL-C_KM-6-TG_, LDL-C_KM-10_, LDL-C_KM-12-TG_, LDL-C_KM-12_, LDL-C_KM-28_, and LDL-C_KM-180_): LDL-C calculated using the N-cell tables with the median TG/VLDL-C ratios derived from our dataset; LDL-C_KO-N_ (LDL-C_KO-6-TG_, LDL-C_KO-10_, LDL-C_KO-12-TG_, LDL-C_KO-12_, and LDL-C_KO-28_): LDL-C calculated using the N-cell tables with the optimal TG/VLDL-C ratios derived from our dataset; NA: not applicable.

*^a^* The values in the table are *p*-values. Statistical significance of differences in overall concordance between two LDL-C estimates was assessed using McNemar’s exact test for correlated proportions.

*^b^* When stratification was based on TG levels alone, rather than combined TG and non–HDL-C levels, the subscript “_TG_” was added, as in LDL-C_KM-N-TG_ or LDL-C_KO-N-TG_.
