# Supplementary material for: Refining the Martin–Hopkins method for estimating low-density lipoprotein cholesterol levels: Median versus optimal TG/VLDL-C ratio
Source: PLoS One. 2025 Jul 3;20(7):e0327169. doi: 10.1371/journal.pone.0327169 (PMC12225850; doi:10.1371/journal.pone.0327169)
Supplement: S11 Table — (DOCX) [file pone.0327169.s012.docx]

|  |  |  | LDL-C_F_ | LDL-C_M-180_ | LDL-C_KO-28_ |
| --- | --- | --- | --- | --- | --- |
| TGs, mg/dL | LDL-C, mg/dL | *n* | C/T (%) | C/T (%) | C/T (%) |
| < 400 |  | 11,930 | 9,500 / 11,930 (79.6) | 9,925 / 11,930 (83.2) | 10,020 / 11,930 (84.0) |
|  | < 70 | 886 | 700 / 964 (72.6) | 675 / 796 (84.8) | 671 / 761 (88.2) |
|  | 70–99 | 3,675 | 3,049 / 3,793 (80.4) | 3,134 / 3,650 (85.9) | 3,122 / 3,569 (87.5) |
|  | 100–129 | 4,218 | 3,371 / 4,158 (81.1) | 3,564 / 4,252 (83.8) | 3,688 / 4,439 (83.1) |
|  | 130–159 | 2,274 | 1,728 / 2,168 (79.7) | 1,863 / 2,330 (80.0) | 1,887 / 2,328 (81.1) |
|  | 160 –189 | 712 | 521 / 677 (77.0) | 556 / 733 (75.9) | 527 / 671 (78.5) |
|  | ≥ 190 | 165 | 131 / 170 (77.1) | 133 / 169 (78.7) | 125 / 162 (77.2) |
| < 150 |  | 8,912 | 7,424 / 8,912 (83.3) | 7,570 / 8,912 (84.9) | 7,611 / 8,912 (85.4) |
|  | < 70 | 732 | 557 / 663 (84.0) | 573 / 683 (83.9) | 563 / 645 (87.3) |
|  | 70–99 | 3,000 | 2,565 / 2,994 (85.7) | 2,632 / 3,044 (86.5) | 2,590 / 2,933 (88.3) |
|  | 100–129 | 3,159 | 2,650 / 3,153 (84.0) | 2,690 / 3,115 (86.4) | 2,802 / 3,312 (84.6) |
|  | 130–159 | 1,491 | 1,226 / 1,540 (79.6) | 1,245 / 1,525 (81.6) | 1,246 / 1,504 (82.8) |
|  | 160–189 | 440 | 351 / 458 (76.6) | 356 / 451 (78.9) | 344 / 432 (79.6) |
|  | ≥ 190 | 90 | 75 / 104 (72.1) | 74 / 94 (78.7) | 66 / 86 (76.7) |
| 150–399 |  | 3,018 | 2,076 / 3,018 (68.8) | 2,355 / 3,018 (78.0) | 2,409 / 3,018 (79.8) |
|  | < 70 | 154 | 143 / 301 (47.5) | 102 / 113 (90.3) | 108 / 116 (93.1) |
|  | 70–99 | 675 | 484 / 799 (60.6) | 502 / 606 (82.8) | 532 / 636 (83.6) |
|  | 100–129 | 1,059 | 721 / 1,005 (71.7) | 874 / 1,137 (76.9) | 886 / 1,127 (78.6) |
|  | 130–159 | 783 | 502 / 628 (79.9) | 618 / 805 (76.8) | 641 / 824 (77.8) |
|  | 160–189 | 272 | 170 / 219 (77.6) | 200 / 282 (70.9) | 183 / 239 (76.6) |
|  | ≥ 190 | 75 | 56 / 66 (84.8) | 59 / 75 (78.7) | 59 / 76 (77.6) |

**Abbreviations:** NCEP–ATP III: National Cholesterol Education Program Adult Treatment Panel III; TG: triglyceride; LDL-C: low-density lipoprotein cholesterol; LDL-C_F_: LDL-C calculated using the Friedewald formula; LDL-C_M-180_: LDL-C calculated using the original 180-cell Martin–Hopkins method proposed by Martin et al. [14]; LDL-C_KO-28_: LDL-C calculated using the 28-cell table (Fig 2) with the optimal ratios of triglycerides to very-low-density lipoprotein cholesterol (TG/VLDL-C) derived from our dataset; C/T: concordant number / total number in each group.
