# Supplementary material for: Refining the Martin–Hopkins method for estimating low-density lipoprotein cholesterol levels: Median versus optimal TG/VLDL-C ratio
Source: PLoS One. 2025 Jul 3;20(7):e0327169. doi: 10.1371/journal.pone.0327169 (PMC12225850; doi:10.1371/journal.pone.0327169)
Supplement: S12 Table — (DOCX) [file pone.0327169.s013.docx]

|  |  | LDL-C_D_, mg/dL *^a^* | | | | | |
| --- | --- | --- | --- | --- | --- | --- | --- |
|  | *n* | < 70 | 70–99 | 100–129 | 130–159 | 160–189 | ≥ 190 |
| LDL-C_F_, mg/dL |  |  |  |  |  |  |  |
| < 70 | 964 | **700 (72.6)** | 264 (27.4) |  |  |  |  |
| 70–99 | 3,793 | 184 ( 4.9) | **3,049 (80.4)** | 599 (14.7) | 1 ( 0.0) |  |  |
| 100–129 | 4,158 | 2 ( 0.0) | 362 ( 8.7) | **3,371 (81.1)** | 422 (10.1) | 1 ( 0.0) |  |
| 130–159 | 2,168 |  |  | 288 (13.3) | **1,728 (79.7)** | 152 ( 7.0) |  |
| 160–189 | 677 |  |  |  | 122 (18.0) | **521 (77.0)** | 34 ( 5.0) |
| ≥ 190 | 170 |  |  |  | 1 ( 0.6) | 38 (22.4) | **165 (77.1)** |
| LDL-C_M-180_, mg/dL |  |  |  |  |  |  |  |
| < 70 | 796 | **675 (84.8)** | 121 (15.1) |  |  |  |  |
| 70–99 | 3,650 | 208 ( 5.7) | **3,134 (85.9)** | 308 ( 8.4) |  |  |  |
| 100–129 | 4,252 | 3 ( 0.1) | 418 ( 9.8) | **3,564 (83.8)** | 267 ( 6.3) |  |  |
| 130–159 | 2,330 |  | 2 ( 0.1) | 344 (14.8) | **1,863 (80.0)** | 121 ( 5.2) |  |
| 160–189 | 733 |  |  | 2 ( 0.3) | 143 (19.5) | **556 (75.9)** | 32 ( 4.4) |
| ≥ 190 | 169 |  |  |  | 1 ( 0.6) | 35 (20.7) | **133 (78.7)** |
| LDL-C_KO-28_, mg/dL |  |  |  |  |  |  |  |
| < 70 | 761 | **671 (88.2)** | 90 (11.8) |  |  |  |  |
| 70–99 | 3,569 | 211 ( 5.9) | **3,122 (87.5)** | 236 ( 6.6) |  |  |  |
| 100–129 | 4,439 | 4 ( 0.1) | 463 (10.4) | **3,688 (83.1)** | 284 ( 6.4) |  |  |
| 130–159 | 2,328 |  |  | 292 (12.5) | **1,887 (81.1)** | 149 ( 6.4) |  |
| 160–189 | 671 |  |  | 2 ( 0.3) | 102 (15.2) | **527 (78.5)** | 40 ( 6.0) |
| ≥ 190 | 162 |  |  |  | 1 ( 0.6) | 36 (22.2) | **125 (77.2)** |

**Abbreviations:** NCEP–ATP III: National Cholesterol Education Program Adult Treatment Panel III; LDL-C: low-density lipoprotein cholesterol; LDL-C_D_: LDL-C directly measured using the homogeneous enzymatic assay; LDL-C_F_: LDL-C calculated using the Friedewald formula; LDL-C_M-180_: LDL-C calculated using the 180-cell Martin–Hopkins method proposed by Martin et al. [14]; LDL-C_KO-28_: LDL-C calculated using the 28-cell table (Fig 2) with the optimal ratios of triglycerides to very-low-density lipoprotein cholesterol (TG/VLDL-C) derived from our dataset.

*^a^* Values are presented as numbers (percentages within each LDL-C_F_ category). Bold text highlights the concordant group between directly measured LDL-C and its estimates according to the NCEP–ATP III guideline classification.
