# Supplementary material for: Refining the Martin–Hopkins method for estimating low-density lipoprotein cholesterol levels: Median versus optimal TG/VLDL-C ratio
Source: PLoS One. 2025 Jul 3;20(7):e0327169. doi: 10.1371/journal.pone.0327169 (PMC12225850; doi:10.1371/journal.pone.0327169)
Supplement: S13 Table — (DOCX) [file pone.0327169.s014.docx]

|  |  | Reclassification using LDL-C_M-180_ *^a^* | | | Reclassification using LDL-C_KO-28_ *^a^* | | |
| --- | --- | --- | --- | --- | --- | --- | --- |
| LDL-C_F_, mg/dL | Classification | Correct | Incorrect | *p*-value *^b^* | Correct | Incorrect | *p*-value *^b^* |
| < 70 | Correct | 533 (80.4) | **24 ( 3.6)** | 0.077 | 535 (80.7) | **22 ( 3.3)** | 0.040 |
| (*n* = 663) | Incorrect | **39 ( 5.9)** | 67 (10.1) |  | **39 ( 5.9)** | 67 (10.1) |  |
| 70–99 | Correct | 2,506 (83.7) | **59 ( 2.0)** | < 0.001 | 2,504 (83.6) | **61 ( 2.0)** | < 0.001 |
| (*n* = 2,994) | Incorrect | **110 ( 3.7)** | 319 (10.7) |  | **135 ( 4.5)** | 294 ( 9.8) |  |
| 100–129 | Correct | 2,561 (81.2) | **89 ( 2.8)** | 0.051 | 2,592 (82.2) | **58 ( 1.8)** | 0.003 |
| (*n* = 3,153) | Incorrect | **118 ( 3.7)** | 385 (12.2) |  | **96 ( 3.0)** | 407 (12.9) |  |
| 130–159 | Correct | 1,196 (77.7) | **30 ( 1.9)** | < 0.001 | 1,167 (75.8) | **59 ( 3.8)** | < 0.001 |
| (*n* = 1,540) | Incorrect | **63 ( 4.1)** | 251 (16.3) |  | **107 ( 6.9)** | 207 (13.4) |  |
| 160–189 | Correct | 343 (74.9) | **8 ( 1.7)** | 0.076 | 331 (72.3) | **20 ( 4.4)** | 0.203 |
| (*n* = 458) | Incorrect | **18 ( 3.9)** | 89 (19.4) |  | **30 ( 6.6)** | 77 (16.8) |  |
| ≥ 190 | Correct | 74 (71.2) | **1 ( 1.0)** | 0.021 | 66 (63.5) | **9 ( 8.7)** | 1.000 |
| (*n* = 104) | Incorrect | **9 ( 8.7)** | 20 (19.2) |  | **9 ( 8.7)** | 20 (19.2) |  |
| Overall | Correct | 7,213 (80.9) | **211 ( 2.4)** | < 0.001 | 7,195 (80.7) | **229 ( 2.6)** | < 0.001 |
| (*n* = 8,912) | Incorrect | **357 ( 4.0)** | 1,131(12.7) |  | **416 ( 4.7)** | 1,072 (12.0) |  |

**Abbreviations:** TG: triglyceride; LDL-C: low-density lipoprotein cholesterol; LDL-C_F_: LDL-C calculated using the Friedewald formula; LDL-C_M-180_: LDL-C calculated using the original 180-cell Martin–Hopkins equation proposed by Martin et al. [14]; LDL-C_KO-28_: LDL-C calculated using the 28-cell table (Fig 2) with the optimal ratios of triglycerides to very-low-density lipoprotein cholesterol (TG/VLDL-C) derived from our dataset.

*^a^* Values are presented as numbers (percentages within each LDL-C_F_ category). Boldfaced values highlight cases in which the classification by LDL-C_M-180_ or LDL-C_KO-28_ differed from that of LDL-C_F_, based on directly measured LDL-C.

*^b^* Statistical significance of the differences in concordance between each LDL-C estimate and LDL-C_F_ was assessed using McNemar’s exact test for correlated proportions.
