# Supplementary material for: Refining the Martin–Hopkins method for estimating low-density lipoprotein cholesterol levels: Median versus optimal TG/VLDL-C ratio
Source: PLoS One. 2025 Jul 3;20(7):e0327169. doi: 10.1371/journal.pone.0327169 (PMC12225850; doi:10.1371/journal.pone.0327169)
Supplement: S14 Table — (DOCX) [file pone.0327169.s015.docx]

|  |  | Reclassification using LDL-C_M-180_ *^a^* | | | Reclassification using LDL-C_KO-28_ *^a^* | | |
| --- | --- | --- | --- | --- | --- | --- | --- |
| LDL-C_F_, mg/dL | Classification | Correct | Incorrect | *p*-value *^b^* | Correct | Incorrect | *p*-value *^b^* |
| < 70 | Correct | 102 (33.9) | **41 (13.6)** | < 0.001 | 108 (35.9) | **35 (11.6)** | < 0.001 |
| (*n* = 301) | Incorrect | **147 (48.8)** | 11 ( 3.7) |  | **150 (49.8)** | 8 ( 2.7) |  |
| 70–99 | Correct | 355 (44.4) | **129 (16.1)** | < 0.001 | 382 (47.8) | **102 (12.8)** | < 0.001 |
| (*n* = 799) | Incorrect | **249 (32.1)** | 66 ( 8.3) |  | **243 (30.4)** | 72 ( 9.0) |  |
| 100–129 | Correct | 625 (62.2) | **96 ( 9.6)** | < 0.001 | 643 (64.0) | **78 ( 7.8)** | < 0.001 |
| (*n* = 1,005) | Incorrect | **150 (14.9)** | 134 (13.3) |  | **147 (14.6)** | 137 (13.6) |  |
| 130–159 | Correct | 468 (74.5) | **34 ( 5.4)** | 0.905 | 481 (76.6) | **21 ( 3.3)** | 0.169 |
| (*n* = 628) | Incorrect | **36 ( 5.7)** | 90 (14.3) |  | **32 ( 5.1)** | 94 (15.0) |  |
| 160–189 | Correct | 164 (74.9) | **6 ( 2.7)** | 0.508 | 150 (68.5) | **20 ( 9.1)** | 0.871 |
| (*n* = 219) | Incorrect | **3 ( 1.4)** | 46 (21.0) |  | **18 ( 8.2)** | 31 (14.2) |  |
| ≥ 190 | Correct | 56 (84.8) | **0 ( 0.0)** | 1.000 | 54 (81.8) | 2 ( 3.0) | 1.000 |
| (*n* = 66) | Incorrect | **0 ( 0.0)** | 10 (15.2) |  | **1 ( 1.5)** | 9 (13.6) |  |
| Overall | Correct | 1,770 (58.6) | **306 (10.1)** | < 0.001 | 1,818 (60.2) | **258 ( 8.5)** | < 0.001 |
| (*n* = 3,018) | Incorrect | **585 (19.4)** | 357 (11.8) |  | **591 (19.6)** | 351 (11.6) |  |

**Abbreviations:** TG: triglyceride; LDL-C: low-density lipoprotein cholesterol; LDL-C_F_: LDL-C calculated using the Friedewald formula; LDL-C_M-180_: LDL-C calculated using the original 180-cell Martin–Hopkins equation proposed by Martin et al. [14]; LDL-C_KO-28_: LDL-C calculated using the 28-cell table (Fig 2) with the optimal ratios of triglycerides to very-low-density lipoprotein cholesterol (TG/VLDL-C) derived from our dataset.

*^a^* Values are presented as numbers (percentages within each LDL-C_F_ category). Boldfaced values highlight cases in which the classification by LDL-C_M-180_ or LDL-C_KO-28_ differed from that of LDL-C_F_, based on directly measured LDL-C.

*^b^* Statistical significance of the differences in concordance between each LDL-C estimate and LDL-C_F_ was assessed using McNemar’s exact test for correlated proportions.
