# Supplementary material for: Refining the Martin–Hopkins method for estimating low-density lipoprotein cholesterol levels: Median versus optimal TG/VLDL-C ratio
Source: PLoS One. 2025 Jul 3;20(7):e0327169. doi: 10.1371/journal.pone.0327169 (PMC12225850; doi:10.1371/journal.pone.0327169)
Supplement: S15 Table — (DOCX) [file pone.0327169.s016.docx]

|  | Estimated LDL-C (LDL-C_E_) *^a^* | | | | | | | | | | | | |
| --- | --- | --- | --- | --- | --- | --- | --- | --- | --- | --- | --- | --- | --- |
| LDL-C_E_ | M-10 | M-180 | KM-6-TG | KO-6-TG | KM-10 | KO-10 | KM-12-TG | KO-12-TG | KM-12 | KO-12 | KM-28 | KO-28 | KM-180 |
| LDL-C_F_ | 0.028 | 0.077 | 0.011 | 0.028 | 0.072 | 0.040 | 0.011 | 0.009 | 0.065 | 0.040 | 0.065 | 0.040 | 0.033 |
| LDL-C_M-10_ | NA | 1 | 1 | 1 | 0.625 | 0.824 | 1 | 0.267 | 1 | 0.824 | 1 | 0.824 | 0.678 |
| LDL-C_M-180_ | 0.077 | NA | 1 | 1 | 0.851 | 0.824 | 1 | 0.227 | 1 | 0.824 | 1 | 0.824 | 0.648 |
| LDL-C_KM-6-TG_ *^b^* | 0.011 | 1 | NA | 1 | 0.791 | 0.856 | 1 | 0.405 | 1 | 0.856 | 0.014 | 0.856 | 0.728 |
| LDL-C_KO-6-TG_ *^b^* | 0.028 | 1 | 1 | NA | 0.625 | 0.824 | 1 | 0.267 | 1 | 0.824 | 1 | 0.824 | 0.678 |
| LDL-C_KM-10_ | 0.072 | 0.625 | 0.851 | 0.791 | NA | 0.454 | 0.791 | 0.143 | 1 | 0.454 | 1 | 0.454 | 0.359 |
| LDL-C_KO-10_ | 0.040 | 0.824 | 0.824 | 0.856 | 0.824 | NA | 0.856 | 0.549 | 0.549 | 1 | 0.549 | 1 | 1 |
| LDL-C_KM-12-TG_ *^b^* | 0.011 | 1 | 1 | 1 | 1 | 0.791 | NA | 0.405 | 1 | 0.856 | 1 | 0.856 | 0.728 |
| LDL-C_KO-12-TG_ *^b^* | 0.009 | 0.267 | 0.227 | 0.405 | 0.267 | 0.143 | 0.549 | NA | 0.18 | 0.549 | 0.18 | 0.549 | 0.832 |
| LDL-C_KM-12_ | 0.065 | 1 | 1 | 1 | 1 | 1 | 0.549 | 1 | NA | 0.549 | 1 | 0.549 | 0.481 |
| LDL-C_KO-12_ | 0.040 | 0.824 | 0.824 | 0.856 | 0.824 | 0.454 | 1 | 0.856 | 0.549 | NA | 0.549 | 1 | 1 |
| LDL-C_KM-28_ | 0.065 | 1 | 1 | 0.014 | 1 | 1 | 0.549 | 1 | 0.18 | 1 | NA | 0.549 | 0.549 |
| LDL-C_KO-28_ | 0.040 | 0.824 | 0.824 | 0.856 | 0.824 | 0.454 | 1 | 0.856 | 0.549 | 0.549 | 1 | NA | 1 |
| LDL-C_KM-180_ | 0.033 | 0.678 | 0.648 | 0.728 | 0.678 | 0.359 | 1 | 0.728 | 0.832 | 0.481 | 1 | 0.549 | NA |

**Abbreviations:** LDL-C: low-density lipoprotein cholesterol; LDL-C_E_: estimated LDL-C; LDL-C_F_: LDL-C calculated using the Friedewald formula; LDL-C_M-N_ (LDL-C_M-10_ and LDL-C_M-180_): LDL-C calculated using the N-cell tables with the median ratios of triglycerides to very-low-density lipoprotein cholesterol (TG/VLDL-C) reported by Martin et al. [14]; LDL-C_KM-N_ (LDL-C_KM-6-TG_, LDL-C_KM-10_, LDL-C_KM-12-TG_, LDL-C_KM-12_, LDL-C_KM-28_, and LDL-C_KM-180_): LDL-C calculated using the N-cell tables with the median TG/VLDL-C ratios derived from our dataset; LDL-C_KO-N_ (LDL-C_KO-6-TG_, LDL-C_KO-10_, LDL-C_KO-12-TG_, LDL-C_KO-12_, and LDL-C_KO-28_): LDL-C calculated using the N-cell tables with the optimal TG/VLDL-C ratios derived from our dataset; NA: not applicable.

*^a^* The values in the table are *p*-values. Statistical significance of differences in overall concordance between two LDL-C estimates was assessed using McNemar’s exact test for correlated proportions.

*^b^* When stratification was based on TG levels alone, rather than combined TG and non–HDL-C levels, the subscript “_TG_” was added, as in LDL-C_KM-N-TG_ or LDL-C_KO-N-TG_.
