# Supplementary material for: Refining the Martin–Hopkins method for estimating low-density lipoprotein cholesterol levels: Median versus optimal TG/VLDL-C ratio
Source: PLoS One. 2025 Jul 3;20(7):e0327169. doi: 10.1371/journal.pone.0327169 (PMC12225850; doi:10.1371/journal.pone.0327169)
Supplement: S16 Table — (DOCX) [file pone.0327169.s017.docx]

|  | Estimated LDL-C (LDL-C_E_) *^a^* | | | | | | | | | | | | |
| --- | --- | --- | --- | --- | --- | --- | --- | --- | --- | --- | --- | --- | --- |
| LDL-C_E_ | M-10 | M-180 | KM-6-TG | KO-6-TG | KM-10 | KO-10 | KM-12-TG | KO-12-TG | KM-12 | KO-12 | KM-28 | KO-28 | KM-180 |
| LDL-C_F_ | <0.001 | <0.001 | <0.001 | <0.001 | <0.001 | <0.001 | <0.001 | <0.001 | <0.001 | <0.001 | <0.001 | <0.001 | <0.001 |
| LDL-C_M-10_ | NA | 0.791 | 0.659 | 0.878 | 0.791 | 0.152 | 0.659 | 0.511 | 0.581 | 0.035 | 0.454 | 0.080 | 0.824 |
| LDL-C_M-180_ | <0.001 | NA | 0.519 | 0.689 | 0.572 | 0.418 | 0.519 | 0.775 | 1 | 0.248 | 0.832 | 0.108 | 0.572 |
| LDL-C_KM-6-TG_ *^b^* | <0.001 | 0.659 | NA | 0.625 | 0.864 | 0.017 | 1 | 0.022 | 0.296 | 0.035 | 0.442 | 0.063 | 0.864 |
| LDL-C_KO-6-TG_ *^b^* | <0.001 | 0.878 | 0.689 | NA | 1 | 0.031 | 0.625 | 0.092 | 0.458 | 0.061 | 0.405 | 0.098 | 1 |
| LDL-C_KM-10_ | <0.001 | 0.791 | 0.572 | 0.864 | NA | 0.006 | 0.864 | 0.230 | 0.063 | 0.007 | 0.146 | 0.047 | 1 |
| LDL-C_KO-10_ | <0.001 | 0.152 | 0.418 | 0.017 | 0.031 | NA | 0.017 | 0.581 | 0.227 | 1 | 0.481 | 0.736 | 0.031 |
| LDL-C_KM-12-TG_ *^b^* | <0.001 | 0.659 | 0.519 | 1 | 0.625 | 0.864 | NA | 0.022 | 0.296 | 0.035 | 0.268 | 0.063 | 0.864 |
| LDL-C_KO-12-TG_ *^b^* | <0.001 | 0.511 | 0.775 | 0.022 | 0.092 | 0.230 | 0.581 | NA | 0.839 | 0.541 | 1 | 0.461 | 0.248 |
| LDL-C_KM-12_ | <0.001 | 0.581 | 1 | 0.296 | 0.458 | 0.063 | 0.227 | 0.296 | NA | 0.109 | 1 | 0.215 | 0.302 |
| LDL-C_KO-12_ | <0.001 | 0.035 | 0.248 | 0.035 | 0.061 | 0.007 | 1 | 0.035 | 0.541 | NA | 0.332 | 0.839 | 0.027 |
| LDL-C_KM-28_ | <0.001 | 0.454 | 0.832 | 0.442 | 0.405 | 0.146 | 0.481 | 0.268 | 1 | 1 | NA | 0.230 | 0.230 |
| LDL-C_KO-28_ | <0.001 | 0.080 | 0.108 | 0.063 | 0.098 | 0.047 | 0.736 | 0.063 | 0.461 | 0.215 | 0.839 | NA | 0.035 |
| LDL-C_KM-180_ | <0.001 | 0.824 | 0.572 | 0.864 | 1 | 1 | 0.031 | 0.864 | 0.248 | 0.302 | 0.027 | 0.230 | NA |

**Abbreviations:** LDL-C: low-density lipoprotein cholesterol; LDL-C_E_: estimated LDL-C; LDL-C_F_: LDL-C calculated using the Friedewald formula; LDL-C_M-N_ (LDL-C_M-10_ and LDL-C_M-180_): LDL-C calculated using the N-cell tables with the median ratios of triglycerides to very-low-density lipoprotein cholesterol (TG/VLDL-C) reported by Martin et al. [14]; LDL-C_KM-N_ (LDL-C_KM-6-TG_, LDL-C_KM-10_, LDL-C_KM-12-TG_, LDL-C_KM-12_, LDL-C_KM-28_, and LDL-C_KM-180_): LDL-C calculated using the N-cell tables with the median TG/VLDL-C ratios derived from our dataset; LDL-C_KO-N_ (LDL-C_KO-6-TG_, LDL-C_KO-10_, LDL-C_KO-12-TG_, LDL-C_KO-12_, and LDL-C_KO-28_): LDL-C calculated using the N-cell tables with the optimal TG/VLDL-C ratios derived from our dataset; NA: not applicable.

*^a^* The values in the table are *p*-values. Statistical significance of differences in overall concordance between two LDL-C estimates was assessed using McNemar’s exact test for correlated proportions.

*^b^* When stratification was based on TG levels alone, rather than combined TG and non–HDL-C levels, the subscript “_TG_” was added, as in LDL-C_KM-N-TG_ or LDL-C_KO-N-TG_.
