# Supplementary material for: Refining the Martin–Hopkins method for estimating low-density lipoprotein cholesterol levels: Median versus optimal TG/VLDL-C ratio
Source: PLoS One. 2025 Jul 3;20(7):e0327169. doi: 10.1371/journal.pone.0327169 (PMC12225850; doi:10.1371/journal.pone.0327169)
Supplement: Attachment — Submitted filename: Response to Reviewers.docx [file pone.0327169.s019.docx]

**Response to Reviewer 1 Comments**

We sincerely appreciate your encouraging and insightful comments, which helped us to better clarify and refine the key points of our manuscript. Below, we repeat your points and address them individually.

**Point 1**: It is a record-based study; thus, using beta quantification for LDL-C estimation is implausible. However, a homogenous method (Sekisui Medical) was employed. In a previous study (10.1373/clinchem.2009.142810), this method failed to achieve the total allowable error (TAE) as per the NCEP guidelines in LDL-C quantification among diseased subjects, though it worked well in normal people. Based on this fact, kindly answer the following points:
(A) How do you justify this method in the absence of the gold standard, as your data must have included diseased subjects?
(B) Kindly mention the total error with imprecision and bias, separately related to your data.

**Response 1 (A)**:

We appreciate the reviewer’s insightful comments regarding the absence of the beta-quantification reference method in our study and the implications of using the Sekisui homogeneous LDL-C assay. We fully acknowledge that beta quantification remains the gold standard reference method for measuring LDL-C. However, due to logistical constraints and the large-scale nature of the Korea National Health and Nutrition Examination Survey (KNHANES), the use of beta quantification was not feasible. Instead, KNHANES employed a homogeneous enzymatic assay (Sekisui Medical), which has been widely adopted in routine clinical laboratories and national health surveys in Korea.

As the reviewer correctly noted, Miller et al. (2010) reported that the Sekisui homogeneous method did not meet the National Cholesterol Education Program (NCEP) total allowable error (TAE) criterion of ±12% in certain diseased populations. Nevertheless, among the homogeneous assays evaluated, it showed one of the lowest total errors (13.5%) and the smallest negative mean bias (–1.7%).

Importantly, a more recent multicenter study by Miida et al. (2012) confirmed that the Sekisui assay demonstrated good agreement with the beta-quantification method and satisfied the NCEP total error criterion (< 12%) in both non-diseased and diseased populations. Specifically, within the diseased group, the reported total errors by triglyceride (TG) level were:

- 11.5% for TG levels < 11.29 mmol/L,
- 11.1% for TG levels < 6.78 mmol/L, and
- 10.8% for TG levels < 4.52 mmol/L.

These findings support the appropriateness of using the Sekisui assay in large-scale, population-based studies like ours.

**Response 1 (B)**:

Thank you for this important request. To address the reviewer’s request regarding the total error and its components, we refer to the results of the multicenter evaluation study by Miida et al. (2012), which assessed the analytical performance of the Sekisui homogeneous enzymatic method across various TG levels and clinical conditions.

Because our analysis was restricted to participants with TG levels < 400 mg/dL (4.52 mmol/L), we cite the corresponding subgroup results for diseased individuals. According to Table 2 of Miida et al. (2012), the analytical performance metrics under these conditions were as follows:

- Imprecision (CV_t_): 4.7%
- Mean bias: 1.0%
- Total error (TE): 10.9%

The total error value of 10.9% falls within the NCEP recommended threshold of < 12%, even in the diseased subgroup. Therefore, considering that our study population included only individuals with TG levels < 400 mg/dL, we believe that the use of the Sekisui assay is analytically justified and reliable for assessing LDL-C estimation methods in this context.

To address this issue, we have added the following clarification to the **Study limitations** section on pages 44–45:

This study has several limitations. First and foremost, we did not use beta quantification, the reference method for LDL-C measurement. Due to the large-scale nature and logistic constraints of the KNHANES, beta quantification was not feasible. Instead, KNHANES employed the Sekisui homogeneous LDL-C assay, which has been widely adopted in national health surveys and routine clinical laboratories across Korea. Although a previous study [39] reported that the Sekisui assay did not meet the NCEP total allowable error (TAE) criterion of ±12% in some diseased populations, more recent evidence from a multicenter study by Miida et al. [40] demonstrated that the assay satisfied the NCEP criteria, with a total error of 10.9%, an imprecision (total coefficient of variation, CV_t_) of 4.7%, and a mean bias of 1.0% in diseased individuals with TG levels < 400 mg/dL—consistent with our study population. Therefore, while we acknowledge the absence of the reference method, we believe that the analytical performance of the Sekisui homogeneous assay justifies its use in this context. Nonetheless, our findings should be interpreted with this limitation in mind.

We hope that this clarification adequately addresses the reviewer’s concern and reinforces the validity of our methodological approach within the practical constraints of a large-scale epidemiological study.

**Point 2**: Include Bland–Altman plots for the concordance study.

**Response 2**:

We thank you for this valuable suggestion. In response, we have included Bland–Altman analyses to assess the agreement between estimated LDL-C (LDL-C_E_) and directly measured LDL-C (LDL-C_D_). This analysis was conducted for three estimation methods: the Friedewald formula (LDL-C_F_), the original 180-cell Martin–Hopkins equation (LDL-C_M-180_), and our optimized 28-cell model (LDL-C_KO-28_). For each method, the bias (i.e., the difference between LDL-C_E_ and LDL-C_D_) was plotted against the mean of the two values, and the mean bias as well as the 95% limits of agreement (mean ± 1.96 SD) were calculated.

These analyses have been incorporated into the **Materials and Methods** (Data Analysis) and **Results** sections, and are illustrated in the newly added **Figure 4**. We specifically highlighted individuals with triglyceride (TG) levels of 300–399 mg/dL (indicated by green dots) because among the Martin–Hopkins-based LDL-C estimates, LDL-C_M-180_ showed the lowest concordance in this TG range. For this subgroup, the mean bias and 95% limits of agreement were: −13.49 ± 28.61 mg/dL for LDL-C_F_, 6.69 ± 26.19 mg/dL for LDL-C_M-180_, and 2.61 ± 25.51 mg/dL for LDL-C_KO-28_. These results demonstrate improved agreement with directly measured LDL-C when using our optimized method.

We believe that the inclusion of Bland–Altman plots provides a valuable visual representation of agreement and reinforces the validity of our findings. Figure 4 has been appended to this response letter for the reviewer’s convenience.

**Changes Made:**

• **Materials and Methods – Data Analysis** (page 11):

Bland–Altman analysis was performed to assess the agreement between LDL-C_E_ and LDL-C_D_. For three estimation methods (LDL-C_F_, LDL-C_M-180_, and LDL-C_KO-28_), the bias (difference between LDL-C_E_ and LDL-C_D_) was plotted against the mean of the two values. The mean bias and the 95% limits of agreement (mean ± 1.96 SD) were calculated to evaluate systematic differences and dispersion across the range of LDL-C values. In addition, model fit and error metrics were assessed for each LDL-C estimation method in comparison with LDL-C_D_, including the coefficient of determination (*R*^2^), mean relative error (MRE), mean absolute error (MAE), and mean squared error (MSE).

• **Results – Figure 4** (pages 23–24):

To further assess the agreement between estimated LDL-C (LDL-C_E_) and directly measured LDL-C (LDL-C_D_), Bland–Altman analyses were conducted for three estimation methods: LDL-C_F_, LDL-C_M-180_, and LDL-C_KO-28_. As shown in Fig 4, each panel displays the bias (i.e., the difference between LDL-C_E_ and LDL-C_D_) plotted against the mean of the two values. The black dashed line indicates the mean bias, while the orange and red dashed lines represent the upper and lower 95% limits of agreement (mean ± 1.96 SD), respectively. Compared to LDL-C_F_, both LDL-C_M-180_ and LDL-C_KO-28_ exhibited reduced bias dispersion and narrower limits of agreement, indicating improved concordance with LDL-C_D_ values. In particular, for individuals with TG levels of 300–399 mg/dL (represented by green dots), the mean bias and 95% limits of agreement (mean ± 1.96 SD) were: −13.49 ± 28.61 mg/dL for LDL-C_F_, 6.69 ± 26.19 mg/dL for LDL-C_M-180_, and 2.61 ± 25.51 mg/dL for LDL-C_KO-28_. These findings provide additional evidence supporting the improved accuracy of the optimized estimation method (LDL-C_KO-28_), especially among individuals with elevated TG levels.

• **Results – Figure 4 legend** (page 24):

**Fig 4. Bland–Altman plots comparing estimated LDL-C values with directly measured LDL-C**. Each panel shows the bias (i.e., the difference between LDL-C_E_ and LDL-C_D_) plotted against the mean of LDL-C_D_ and LDL-C_E_. The black dashed line represents the mean bias, while the orange and red dashed lines indicate the upper and lower 95% limit of agreement (mean ± 1.96 SD), respectively. Panels (A), (B), and (C) correspond to LDL-C_F_, LDL-C_M-180_, and LDL-C_KO-28_, respectively. These plots illustrate differences in bias magnitude and dispersion, reflecting the degree of agreement between each LDL-C_E_ and LDL-C_D_. Green dots represent individuals with TG levels of 300–399 mg/dL. **Abbreviations:** LDL-C: low-density lipoprotein cholesterol; TG: triglyceride; LDL-C_E_: estimated LDL-C; LDL-C_D_: LDL-C directly measured using a homogeneous enzymatic assay; LDL-C_F_: LDL-C calculated using the Friedewald formula; LDL-C_M-180_: LDL-C calculated using the original 180-cell Martin–Hopkins equation proposed by Martin et al. [14]; LDL-C_KO-28_: LDL-C calculated using the 28-cell table (Fig 2) with the optimal ratios of TG/VLDL-C (triglycerides to very-low-density lipoprotein cholesterol) derived from our dataset; SD: standard deviation.

We hope this addition meets the reviewer’s request and strengthens the manuscript by offering a robust graphical assessment of estimation accuracy.

**(A) LDL-C_F_ versus LDL-C_D_	(B) LDL-C_M-180_ versus LDL-C_D_	(C) LDL-C_KO-28_ versus LDL-C_D_**

**Fig 4. Bland–Altman plots comparing estimated LDL-C values with directly measured LDL-C**. Each panel shows the bias (i.e., the difference between LDL-C_E_ and LDL-C_D_) plotted against the mean of LDL-C_D_ and LDL-C_E_. The back dashed line represents the mean bias, while the orange and red dashed lines indicate the upper and lower 95% limit of agreement (mean ± 1.96 SD), respectively. Panels (A), (B), and (C) correspond to LDL-C_F_, LDL-C_M-180_, and LDL-C_KO-28_, respectively. These plots illustrate differences in bias magnitude and dispersion, reflecting the degree of agreement between each LDL-C_E_ and LDL-C_D_. Green dots represent individuals with TG levels of 300-399 mg/dL. **Abbreviations:** LDL-C: low-density lipoprotein cholesterol; TG: triglyceride; LDL-C_E_: estimated LDL-C; LDL-C_D_: LDL-C directly measured using a homogeneous enzyme assay; LDL-C_F_: LDL-C calculated using the Friedewald formula; LDL-C_M-180_: LDL-C calculated using the original 180-cell Martin–Hopkins equation proposed by Martin et al. [14]; LDL-C_KO-28_: LDL-C calculated using the 28-cell table (Fig 2) with the optimal ratios of triglycerides to very-low-density lipoprotein cholesterol (TG/VLDL-C) derived from our dataset; SD: standard deviation.

**Response to Reviewer 2 Comments**

Thank you very much for your encouragement and thoughtful comments, which stimulated us to rethink the key issues more clearly. Below, we repeat your points and address them individually.

**Point 1**: In the abstract, it is unclear what the connection is between the two methods and whether they are both for low TG levels. I recommend parsing that out more explicitly, as well as the strengths of the Martin–Hopkins method. For example, when it is stated that the method is successful in estimating very-low-density lipoprotein cholesterol, it is unclear what that is compared to 400 mg/dL. Later in the manuscript, it seems that very-low-density lipoprotein cholesterol is referring to lower levels, less than 70-79 mg/dL. I think it would be helpful to delineate that distinction more clearly early on.

**Response 1**:

We appreciate the reviewer’s thoughtful comments. In response, we have revised the manuscript to (1) clarify the performance of LDL-C estimation methods by triglyceride (TG) levels, (2) explicitly define VLDL-C to avoid potential confusion, and (3) enhance the concordance analysis across LDL-C treatment thresholds.

**1. Clarifying performance by TG levels**

In the previous manuscript, TG levels were stratified into six categories (Table 4), and reported that the original 180-cell Martin–Hopkins equation (LDL-C_M-180_) consistently outperformed the Friedewald formula (LDL-C_F_), particularly at higher TG levels.

To better clarify differences in concordance at lower TG levels, we have now added **Figure 3** to the Results section. This figure compares the concordance of LDL-C estimates, stratified into two TG categories: < 150 mg/dL and 150–399 mg/dL. The analysis included not only LDL-C_F_ and LDL-C_M-180_, but also three optimized Martin–Hopkins estimates—LDL-C_KO-6_, LDL-C_KO-12_, and LDL-C_KO-28_—calculated using optimal TG/VLDL-C ratios derived from our dataset. For TG levels < 150 mg/dL, the Martin–Hopkins estimates yielded statistically significantly higher concordance than LDL-C_F_, but the improvement was modest, at around 2%. In contrast, for TG levels of 150–399 mg/dL, the improvements were substantial, with concordance differences reaching approximately 10%.

**2. Clarification of VLDL-C definition**

Regarding the interpretation of VLDL-C, we acknowledge the ambiguity and have revised the manuscript to clarify that VLDL-C in our study does not refer to low LDL-C levels. Instead, it represents a residual cholesterol component calculated by subtracting HDL-C and LDL-C from total cholesterol (i.e., VLDL-C = non–HDL-C − LDL-C). This definition is consistent with standard practice in lipid profiling practice, where VLDL-C represents cholesterol carried by remnant lipoproteins, not a subclass of LDL-C. This clarification is now clearly presented in the **Materials and Methods** section.

**3. Enhanced concordance by LDL-C treatment thresholds**

We also refined our stratified concordance analysis based on estimated LDL-C thresholds as defined by the NCEP-ATP III guideline (< 70, 70–99, 100–129, 130–159, 160–189, ≥ 190 mg/dL). In **Fig 5**, we compared three LDL-C estimates—LDL-C_F_, LDL-C_M-180_, and LDL-C_KO-28_—against directly measured LDL-C within treatment categories, stratified by TG levels: < 150 mg/dL and 150–399 mg/dL. These analyses provide a more explicit understanding of when and where the Martin–Hopkins method offers the greatest benefit.

We hope that these revisions adequately address the reviewer’s concerns and enhance the clarity and clinical relevance of our findings. The revised figures have been incorporated into the manuscript and are also appended to this response letter for your convenience.

**Changes Made:**

• **Abstract – Results** (pages 2–3): We have added a sentence clarifying concordance differences by TG level to better reflect the stratified performance of the estimation methods. Given the word limit, the revision was restricted to a single sentence.

**Results**

The Martin–Hopkins method showed significantly higher concordance than the Friedewald formula for TG levels < 400 mg/dL (79.6% for LDL-C_F_ vs. 83.2% for LDL-C_M-180_, *p* < 0.001). Concordance improved by less than 2% for TG levels < 150 mg/dL (83.3% vs. 84.9%), but by approximately 10% for TG levels of 150–399 mg/dL (68.8% vs. 78.0%). The largest discrepancy was observed in classifying LDL-C levels < 70 mg/dL among individuals with TG levels of 150–399 mg/dL (47.5% for LDL-C_F_ vs. 90.3% for LDL-C_M-180_). However, the overall concordance differed only modestly between the 10-cell and 180-cell Martin–Hopkins equations (82.8% for LDL-C_M-10_ vs. 83.2% for LDL-C_M-180_, a difference of 0.4%), indicating only a marginal benefit despite the substantial increase in the number of strata. Using optimal TG/VLDL-C ratios increased overall concordance compared to median ratios within the same stratification, with LDL-C_KO-N_ estimates outperforming their LDL-C_KM-N_ counterparts. However, this improvement was not statistically significant in LDL-C estimates derived from TG-only stratification.

• **Materials and Methods – Lipid measurements and LDL-C estimation** (page 8): To address the confusion regarding the interpretation of VLDL-C, we revised the manuscript to clearly define its calculation.

Blood samples were collected from each participant’s antecubital vein after an overnight fast of at least 8 hours. Serum lipid concentrations were directly measured using a homogeneous enzymatic assay with an automated analyzer (Hitachi Automatic Analyzer 7600, Hitachi, Tokyo, Japan). The measurements included TC (Pureauto S CHO-N; Sekisui Medical, Tokyo, Japan), HDL-C (Cholestest N HDL; Sekisui Medical), LDL-C (Cholestest N LDL; Sekisui Medical) and triglycerides (Pureauto S TG-N; Sekisui Medical). non–HDL-C was calculated as TC minus HDL-C, and VLDL-C as non–HDL-C minus LDL-C_D_.

• **Results – Figure 3** (pages 21–23): We have added Figure 3 to compare the concordance of LDL-C estimates, stratified by TG levels (< 150 mg/dL and 150–399 mg/dL), with detailed descriptions of the observed differences provided.

Fig 3 illustrates the concordance rates of LDL-C estimates calculated using the Friedewald and Martin–Hopkins methods, compared with directly measured LDL-C, based on the NCEP-ATP III guideline classification. The results are stratified by TG levels, with Fig 3A showing concordance for TG levels < 150 mg/dL and Fig 3B for TG levels of 150–399 mg/dL. LDL-C estimates based on the Martin–Hopkins method included not only LDL-C_M-180_, calculated using the original 180-cell Martin–Hopkins equation, but also LDL-C_KO-6_, LDL-C_KO-12_, and LDL-C_KO-28_, calculated using the optimal TG/VLDL-C ratios derived from our dataset. The statistical significance of differences in concordance between these LDL-C estimates is detailed in S9 and S10 Tables.

Among individuals with TG levels < 150 mg/dL (Fig 3A), all four Martin–Hopkins-based estimates showed statistically significantly higher concordance than the Friedewald-calculated LDL-C (LDL-C_F_); however, the magnitude of improvement was modest, with increases of approximately 2%. In contrast, among individuals with TG levels of 150–399 mg/dL (Fig 3B), each of the Martin–Hopkins-based estimates demonstrated a substantial improvement over LDL-C_F_, with concordance higher by approximately 10% (*p* < 0.001 for all comparisons).

• **Results – Figure 3 legend** (page 22):

**Fig 3. Comparison of concordance rates between Friedewald and Martin–Hopkins LDL-C estimates by TG levels: < 150 mg/dL and 150–399 mg/dL.** Panel (A) shows results for individuals with TG levels < 150 mg/dL, and Panel (B) for those with TG levels of 150–399 mg/dL. Bars represent concordance rates ± 95% confidence intervals for each LDL-C estimate. From left to right, bars correspond to the following LDL-C estimates: LDL-C_F_, LDL-C_M-180_, LDL-C_KO-6_, LDL-C_KO-12_, and LDL-C_KO-28_. * Bars labeled with the same letters indicate no statistically significant difference in concordance between the corresponding LDL-C estimates (*α* = 0.05), based on pairwise comparisons using McNemar’s exact test for correlated proportions (see S9 and S10 Tables). The 95% confidence intervals for concordance rates were calculated using the Clopper–Pearson exact method. **Abbreviations:** NCEP-ATP III: National Cholesterol Education Program Adult Treatment Panel III; TG: triglyceride; LDL-C: low-density lipoprotein cholesterol; LDL-C_F_: LDL-C calculated using the Friedewald formula; LDL-C_M-180_: LDL-C calculated using the original 180-cell Martin–Hopkins equation proposed by Martin et al. [14]; LDL-C_KO-N_ (LDL-C_KO-6-TG_, LDL-C_KO-12_, and LDL-C_KO-28_): LDL-C calculated using the N-cell tables with the optimal ratios of triglycerides to very-low-density lipoprotein cholesterol (TG/VLDL-C) derived from our dataset.

• **Results – Fig 5** (pages 24–26): We have enhanced stratified concordance analysis across LDL-C treatment categories, stratified by TG levels: < 150 mg/dL and 150–399 mg/dL. This allowed for clearer identification of method-specific strengths across LDL-C ranges.

We compared the concordance rates of three LDL-C estimates—LDL-C_F_, LDL-C_M-180_, and LDL-C_KO-28_—with directly measured LDL-C across estimated LDL-C treatment categories defined by the NCEP-ATP III guideline, stratified by TG levels: < 150 mg/dL and 150–399 mg/dL (Fig 5). In individuals with TG levels < 150 mg/dL, the concordance rates for LDL-C_F_ tended to decrease as LDL-C_F_ levels increased, ranging from 84.0% for LDL-C_F_ < 70 mg/dL to 72.1% for LDL-C_F_ ≥ 190 mg/dL. In contrast, among those with TG levels of 150–399 mg/dL, the concordance rates for LDL-C_F_ showed an increasing trend with rising LDL-C_F_ levels, from 47.5% for LDL-C_F_ < 70 mg/dL to 84.8% for LDL-C_F_ ≥ 190 mg/dL (S11 Table for details).

Compared to LDL-C_F_, improvements in concordance for LDL-C_M-180_ and LDL-C_KO-28_ were more pronounced in classifying lower LDL-C levels among individuals with TG levels of 150–399 mg/dL (Fig 5B). The most substantial enhancement in concordance was observed in classifying LDL-C < 70 mg/dL, where concordance increased from 47.5% with LDL-C_F_ to 90.3% and 93.1% with LDL-C_M-180_ and LDL-C_KO-28_, respectively. However, in LDL-C treatment categories ≥ 130 mg/dL within the same TG range, LDL-C_M-180_ and LDL-C_KO-28_ showed lower concordance than LDL-C_F_.

• **Results – Figure 5 legend** (page 25):

**Fig 5. Concordance in the NCEP-ATP III guideline classification: Friedewald vs. Martin–Hopkins LDL-C estimates.** The figure compares concordance rates for LDL-C estimates stratified by TG levels: < 150 mg/dL (A) and 150–399 mg/dL (B). The LDL-C estimates include LDL-C_F_ (gray), LDL-C_M-180_ (deep blue), and LDL-C_KO-28_ (blue). The bars indicate concordance rates for each estimated LDL-C category according to the NCEP-ATP III guideline classification, highlighting differences in concordance among the methods. **Abbreviations:** NCEP-ATP III: National Cholesterol Education Program Adult Treatment Panel III; LDL-C: low-density lipoprotein cholesterol; TG: triglyceride; LDL-C_F_: LDL-C calculated using the Friedewald formula; LDL-C_M-180_: LDL-C calculated using the original 180-cell Martin–Hopkins equation proposed by Martin et al. [14]; LDL-C_KO-28_: LDL-C calculated using the 28-cell table (Fig 2) with the optimal ratios of triglycerides to very-low-density lipoprotein cholesterol (TG/VLDL-C) derived from our dataset.

**(A) Concordance in individuals with TG < 150 mg/dL (B) Concordance in individuals with TG levels of 150-399 mg/dL**

**Fig 3. Comparison of concordance rates between Friedewald and Martin–Hopkins LDL-C estimates by TG levels: < 150 mg/dL and 150–399 mg/dL.** Panel (A) shows results for individuals with TG levels < 150 mg/dL, and Panel (B) for those with TG levels of 150–399 mg/dL. Bars represent concordance rates ± 95% confidence intervals for LDL-C estimates. From left to right, bars correspond to the following LDL-C estimates: LDL-C_F_, LDL-C_M-180_, LDL-C_KO-6_, LDL-C_KO-12_, and LDL-C_KO-28_. * Bars labeled with the same letters indicate that no statistically significant difference in concordance between LDL-C estimates (*α* = 0.05), based on pairwise comparisons using McNemar’s exact test for correlated proportions (see S9 and S10 Tables). The 95% confidence intervals for concordance rates were calculated using the Clopper–Pearson exact method. **Abbreviations:** NCEP-ATP III: National Cholesterol Education Program Adult Treatment Panel III; TG: triglyceride; LDL-C: low-density lipoprotein cholesterol; LDL-C_F_: LDL-C calculated using the Friedewald formula; LDL-C_M-180_: LDL-C calculated using the original 180-cell Martin–Hopkins equation proposed by Martin et al. [14]; LDL-C_KO-N_ (LDL-C_KO-6-TG_, LDL-C_KO-12_, and LDL-C_KO-28_): LDL-C calculated using the N-cell tables with the optimal ratios of triglycerides to very-low-density lipoprotein cholesterol (TG/VLDL-C) ratios derived from our dataset.

**(A) TG levels < 150 mg/dL	(B) TG levels of 150-399 mg/dL**

**Fig 5. Concordance in the NCEP-ATP III guideline classification: Friedewald vs. Martin–Hopkins LDL-C estimates.** The figure compares concordance rates for LDL-C estimates stratified by TG levels: < 150 mg/dL (A) and 150–399 mg/dL (B). The LDL-C estimates include LDL-C_F_ (gray), LDL-C_M-180_ (deep blue), and LDL-C_KO-28_ (blue). The bars indicate concordance rates for each estimated LDL-C category according to the NCEP-ATP III guideline classification, highlighting differences in concordance among the methods. **Abbreviations:** NCEP-ATP III: National Cholesterol Education Program Adult Treatment Panel III; LDL-C: low-density lipoprotein cholesterol; TG: triglyceride; LDL-C_F_: LDL-C calculated using the Friedewald formula; LDL-C_M-180_: LDL-C calculated using the original 180-cell Martin–Hopkins equation proposed by Martin et al. [14]; LDL-C_KO-28_: LDL-C calculated using the 28-cell table (Fig 2) with the optimal ratios of triglycerides to very-low-density lipoprotein cholesterol (TG/VLDL-C) derived from our dataset.

**Point 2**: When showing the formulas for Friedewald and Martin–Hopkins, it is states "where, AF_N is an adjustable factor...", remove the comma, so it is "where AF_N is an adjustable factor."

**Response 2**:

Thank you very much for pointing out the wording issue. We have revised the sentence as suggested. The correction appears on pages 8-9 as follows:

Martin–Hopkins LDL-C (LDL-C_M-N_) was calculated using the following equation [14]:

LDL-C_M-N_ = (non–HDL-C) – (triglycerides / AF_N_ in mg/dL)

where AF_N_ is an adjustable factor determined as the median TG/VLDL-C ratio based on N-cell stratification.

**Point 3**: In the results, it is states "Table 1 summarized" - perhaps it should be "Table 1 summarizes"? Keeping it consistent that the analyses performed are described in the past, but the tables are currently showing results.

**Response 3**:

We appreciate your helpful suggestion regarding verb usage. We have made the correction as recommended. The revision appears on page 12 as follows:

**Table 1** summarizes the sex, age, and lipid characteristics of the study population**.** Population 1 (*n* = 11,930), which was used to validate LDL-C estimates, included 1,500 individuals (12.6%) with TG levels of 200–399 mg/dL.

**Response to Reviewer 3 Comments**

Thank you for your thoughtful and constructive feedback. Your comments prompted us to reflect more deeply on several core aspects of the study. We repeat your points and address them individually below.

**Point 1**: Can the authors discuss the applicability of the findings for global population since this only use Korean population)?

**Response 1**:

We appreciate the reviewer’s important question regarding the generalizability of our findings beyond the Korean population. In response, we have added a dedicated section to address this issue, highlighting several factors that influence the applicability of our approach to other populations.

First, we acknowledge that both ethnic and assay-specific factors may affect lipid profiles and, consequently, the performance of LDL-C estimation equations. Although our strata-specific TG/VLDL-C ratios were derived from a Korean cohort, the methodology itself is not inherently population-specific. Rather, it can be adapted to any population with sufficient lipid profile data and directly measured LDL-C values, allowing laboratories to derive their own optimal ratios from local datasets.

Importantly, we emphasize that our approach does not merely recalibrate the original Martin–Hopkins equation but constitutes a distinct model that optimizes TG/VLDL-C ratios for improved concordance with directly measured LDL-C. For example, despite using fewer stratification cells, the 28-cell optimized LDL-C_KO-28_ achieved slightly higher concordance (84.0%) than the 180-cell median-based LDL-C_KM-180_ (83.7%) among individuals with TG levels < 400 mg/dL.

We also address issues related to LDL-C measurement methods. Our study used a homogeneous enzyme assay, whereas the study by Martin et al. (2013) employed the Vertical Auto Profile (VAP) technique, which fractionates lipoproteins differently. Such methodological differences can substantially affect TG/VLDL-C ratio estimates and should be taken into account when applying or generalizing findings across different clinical laboratories.

Finally, we stress the need for external validation in diverse populations before applying the optimized ratios globally. Nevertheless, our modeling approach offers a scalable framework that other laboratories can implement using their own datasets. These clarifications have been added to the **Discussion** section on pages 42–43 as follows:

**Distinct modeling approach and generalizability**

Although the original Martin–Hopkins method was developed using a large U.S. cohort (*n* > 900,000), our study—based on a Korean cohort (*n* = 18,322)—demonstrated that population-specific optimization can yield superior performance. Importantly, our approach does not merely recalibrate the original Martin–Hopkins equation but represents a distinct model that optimizes TG/VLDL-C ratios to enhance concordance with directly measured LDL-C values. For example, despite employing fewer stratification cells, the 28-cell optimized LDL-C_KO-28_ achieved slightly higher concordance (84.0%) than the 180-cell median-based LDL-C_KM-180_ (83.7%) among individuals with TG levels < 400 mg/dL (Table 4). This finding suggests that careful optimization can outperform more granular stratification schemes based on median values, thereby enabling the use of simpler, lower-resolution stratifications.

Such models may offer greater flexibility for incorporating novel, yet potentially performance-enhancing variables. As larger and more diverse datasets become available, the optimized approach can be extended to include additional parameters (e.g., HDL-C, obesity) beyond TG and non–HDL-C, further improving the accuracy of LDL-C estimation.

**Assay-specific and ethnic considerations**

LDL-C measurement techniques may influence the TG/VLDL-C ratios derived for estimation. Our study employed a homogeneous enzymatic assay, while the original Martin–Hopkins method was developed using the VAP technique, which separates lipoprotein fractions through a different process. These methodological differences can substantially impact the estimation of TG/VLDL-C ratios and, in turn, affect the performance and transferability of estimation methods.

This highlights the need for assay-specific recalibration when applying such models in diverse laboratory settings. In addition, ethnic and genetic factors, along with lifestyle and dietary patterns, may contribute to lipid distribution and TG/VLDL-C ratios across populations. While our optimized model improved concordance in a Korean cohort, external validation in diverse ethnic groups and clinical settings is essential to ensure broader applicability and generalizability across populations.

We hope that this revision appropriately addresses the reviewer’s concern and provides a balanced discussion of both the strengths and the limitations of our findings in the context of global applicability.

**Point 2**: Can the authors add a discussion section on how the improved concordance would affect patient classification or treatment decisions—e.g., how many patients would move across clinical thresholds?

**Response 2**:

We appreciate this thoughtful suggestion regarding the practical relevance of the improved concordance. In response, we have added a new reclassification analysis to evaluate how the improved concordance observed in LDL-C_M-180_ and LDL-C_KO-28_ could influence patient classification and potential treatment decisions.

Specifically, we assessed the proportion of individuals whose LDL-C-based treatment categories—defined by the NCEP-ATP III guideline—would change when using these methods compared to the Friedewald formula (LDL-C_F_), and whether such reclassifications were correct or incorrect relative to directly measured LDL-C.

This new analysis is described in the **Materials and Methods** section, and the results are presented in **Table 5** and **Figure 7**, with further details provided in Supplementary Tables S13 and S14. In addition, we have included a new subsection in the **Discussion**, addressing the clinical implications of these findings.

The results indicate that a substantial proportion of individuals—particularly those with lower LDL-C_F_ values and triglyceride levels of 150–399 mg/dL—would be correctly reclassified using the refined Martin–Hopkins method. This has important implications for more accurate identification of patients eligible for lipid-lowering therapy or more intensive clinical follow-up.

We hope this addition satisfies the reviewer’s request by demonstrating the real-world clinical relevance of improved concordance and its potential impact on guideline-based treatment decisions. The revised figure and tables have been incorporated into the manuscript and are also appended to this response letter for your convenience.

**Changes Made:**

• **Materials and Methods – Data analysis** (page 10): A new paragraph describing the reclassification analysis has been added.

Reclassification was defined as a shift in the guideline-based treatment category when using an alternative LDL-C estimation method or directly measured LDL-C, either upward (to a higher category) or downward (to a lower category), compared with LDL-C_F_. The correctness of each reclassification, whether correct or incorrect, was evaluated against directly measured LDL-C. To evaluate the clinical implications of LDL-C estimation methods, a reclassification analysis was conducted to quantify the improvement of the Martin–Hopkins method over the Friedewald formula in correctly assigning individuals to treatment categories based on LDL-C_D_.

• **Results – Table 5** (page 28): A summary paragraph for reclassification performance has been included.

Table 5 presents the reclassification outcomes of LDL-C_M-180_ and LDL-C_KO-28_ across treatment categories initially assigned by LDL-C_F_ in individuals with TG levels < 400 mg/dL, evaluated against directly measured LDL-C. Among individuals with LDL-C_F_ < 70 mg/dL, 19.3% and 19.6% were correctly reclassified to LDL-C ≥ 70 mg/dL using LDL-C_M-180_ and LDL-C_KO-28_, respectively. However, 6.7% and 5.9% were incorrectly reclassified upward to LDL-C ≥ 70 mg/dL, despite being correctly classified as LDL-C < 70 mg/dL by LDL-C_F_. For all LDL-C_F_ categories below 160 mg/dL, both methods yielded more correct than incorrect reclassifications, leading to statistically significant improvements in classification accuracy compared to LDL-C_F_ (*p* < 0.01 for all comparisons). Overall, LDL-C_KO-28_ demonstrated slightly superior performance to LDL-C_M-180_, with a higher rate of correct reclassifications (8.4% vs. 7.9%) and a lower rate of incorrect reclassifications (4.1% vs. 4.3%).

• **Results – Figure 7** (pages 29–31): Detailed explanations of the stratified reclassification outcomes have been added.

Fig 7 illustrates the proportions of correct (blue) and incorrect (gray) reclassifications using LDL-C_M-180_ and LDL-C_KO-28_, relative to treatment categories initially assigned by LDL-C_F_, according to the NCEP-ATP III guideline. For both methods, reclassification was more frequent among individuals with higher TG levels (Fig 7A and 7B). In particular, for TG levels of 200–299 and 300–399 mg/dL, the proportion of correct reclassifications greatly exceeded that of incorrect ones for both methods, compared to the TG < 150 mg/dL subgroups. Notably, in the highest TG category (300–399 mg/dL), LDL-C_M-180_ exhibited a slightly higher rate of correct reclassification than LDL-C_KO-28_ (42.3% vs. 39.4%), but also a substantially higher rate of incorrect reclassification (20.9% vs. 12.2%).

Fig 7C–7F present reclassification outcomes across treatment categories based on LDL-C_F_, stratified by TG levels. Compared to individuals with TG levels < 150 mg/dL (Fig 7C and 7D), reclassification was markedly more prevalent among those with TG levels of 150–399 mg/dL (Fig 7E and 7F), particularly within lower LDL-C_F_ categories. Among individuals with LDL-C_F_ < 70 mg/dL and TG levels of 150–399 mg/dL, 48.8% and 49.8% were correctly reclassified by LDL-C_M-180_ and LDL-C_KO-28_, respectively. However, 13.6% and 11.6% were incorrectly reclassified by LDL-C_M-180_ and LDL-C_KO-28_, despite being correctly classified as < 70 mg/dL by LDL-C_F_. For LDL-C_F_ categories < 130 mg/dL, both methods yielded significantly more correct than incorrect reclassifications, whereas for LDL-C_F_ categories ≥ 130 mg/dL, the rates of correct and incorrect reclassification were similar and no statistically significant improvement over LDL-C_F_ was observed (see S14 Table).

• **Results – Figure 7 legend** (page 30):

**Fig 7. Correct and incorrect reclassification using LDL-C_M-180_ and LDL-C_KO-28_ compared with LDL-C_F_.** Reclassification was defined as a shift in the NCEP-ATP III guideline-based treatment category initially assigned by LDL-C_F_ when using either LDL-C_M-180_ or LDL-C_KO-28_. The correctness of reclassification was evaluated against directly measured LDL-C, and each panel illustrates the proportions of correct (blue) and incorrect (gray) reclassifications. Panel (A) and Panel (B) show reclassification by triglyceride levels (< 50, 50–99, 100–149, 150–199, 200–299, and 300–399 mg/dL) using LDL-C_M-180_ and LDL-C_KO-28_, respectively. Panel (C) and (D) show reclassification by LDL-C_F_ treatment categories among individuals with TG levels <150 mg/dL, using LDL-C_M-180_ and LDL-C_KO-28_, respectively. Panel (E) and (F) present the same comparisons for individuals with TG levels of 150–399 mg/dL. **Abbreviations:** NCEP-ATP III: National Cholesterol Education Program Adult Treatment Panel III; LDL-C: low-density lipoprotein cholesterol; LDL-C_F_: LDL-C calculated using the Friedewald formula; LDL-C_M-180_: LDL-C calculated using the original 180-cell Martin–Hopkins equation proposed by Martin et al. [14]; LDL-C_KO-28_: LDL-C calculated using the 28-cell table (Fig 2) with the optimal ratios of triglycerides to very-low-density lipoprotein cholesterol (TG/VLDL-C) derived from our dataset.

• **Discussion – LDL-C risk reclassification and implications** (pages 41–42): A new subsection discussing the clinical implications of LDL-C risk reclassification has been added.

**LDL-C risk reclassification and implications**

Our study demonstrates that refining the stratification-based Martin–Hopkins method by applying optimal TG/VLDL-C ratios can improve concordance with directly measured LDL-C categories. To address the clinical relevance of this improvement, we conducted a reclassification analysis to examine how many individuals would move across clinical decision thresholds when using either LDL-C_M-180_ or LDL-C_KO-28_ in place of the Friedewald formula (LDL-C_F_).

Reclassification was particularly notable in individuals with LDL-C_F_ < 70 mg/dL, where 19.3% (LDL-C_M-180_) and 19.6% (LDL-C_KO-28_) were correctly reclassified to LDL-C ≥ 70 mg/dL (Table 5), suggesting potential underestimation by LDL-C_F_ that could affect pharmacologic treatment eligibility. This trend was more pronounced in individuals with TG levels of 150–399 mg/dL, a group prone to misclassification, where nearly half were correctly reclassified (48.8% and 49.8%, respectively), and only 13.6% and 11.6% were incorrectly shifted upward (Fig 7E and 7F). Among individuals with LDL-C_F_ categories < 130 mg/dL and TG levels of 150–399 mg/dL, both methods resulted in significantly more correct than incorrect reclassifications, leading to statistically significant improvements in classification accuracy compared to LDL-C_F_ (S14 Table).

These findings underscore the importance of employing more accurate LDL-C estimation methods, particularly for patients with low LDL-C_F_ and elevated TG levels. Implementing LDL-C_M-180_ and LDL-C_KO-28_ may enable clinicians to more appropriately identify patients who require lipid-lowering therapy or closer clinical follow-up. This improvement in classification accuracy is not only statistically significant but also clinically meaningful.

**Table 5. Reclassification outcomes using LDL-C_M-180_ and LDL-C_KO-28_ across treatment categories assigned by LDL-C_F_, as evaluated against directly measured LDL-C in individuals with triglyceride levels < 400 mg/dL.**

|  |  | Reclassification using LDL-C_M-180_ *^a^* | | | Reclassification using LDL-C_KO-28_ *^a^* | | |
| --- | --- | --- | --- | --- | --- | --- | --- |
| LDL-C_F_, mg/dL | Classification | Correct | Incorrect | *p*-value *^b^* | Correct | Incorrect | *p*-value *^b^* |
| < 70 | Correct | 635 (65.9) | **65 ( 6.7)** | < 0.001 | 643 (66.7) | **57 ( 5.9)** | < 0.001 |
| (*n* = 964) | Incorrect | **186 (19.3)** | 78 ( 8.1) |  | **189 (19.6)** | 75 ( 7.8) |  |
| 70–99 | Correct | 2,861 (75.4) | **188 ( 5.0)** | < 0.001 | 2,886 (76.1) | **163 ( 4.3)** | < 0.001 |
| (*n* = 3,793) | Incorrect | **359 ( 9.5)** | 385 (10.2) |  | **378 (10.0)** | 366 ( 9.6) |  |
| 100–129 | Correct | 3,186 (76.6) | **185 ( 4.4)** | < 0.001 | 3,235 (77.8) | **136 ( 3.3)** | < 0.001 |
| (*n* = 4,158) | Incorrect | **268 ( 6.4)** | 519 (12.5) |  | **243 ( 5.8)** | 544 (13.1) |  |
| 130–159 | Correct | 1,664 (76.8) | **64 ( 3.0)** | 0.008 | 1648 (76.0) | **80 ( 3.7)** | < 0.001 |
| (*n* = 2,168) | Incorrect | **99 ( 4.6)** | 341 (15.7) |  | **139 ( 6.4)** | 301 (13.9) |  |
| 160–189 | Correct | 507 (74.9) | **14 ( 2.1)** | 0.311 | 481 (71.0) | **40 ( 5.9)** | 0.456 |
| (*n* = 677) | Incorrect | **21 ( 3.1)** | 135 (19.9) |  | **48 ( 7.1)** | 108 (16.0) |  |
| ≥ 190 | Correct | 130 (76.5) | **1 ( 0.6)** | 0.021 | 120 (70.6) | **11 ( 6.5)** | 1.000 |
| (*n* = 170) | Incorrect | **9 ( 5.3)** | 30 (17.6) |  | **10 ( 5.9)** | 29 (17.1) |  |
| Overall | Correct | 8,983 (75.3) | **517 ( 4.3)** | < 0.001 | 9,013 (75.5) | 487 ( 4.1) | < 0.001 |
| (*n* = 11,930) | Incorrect | **942 ( 7.9)** | 1,488 (12.5) |  | **1,007 ( 8.4)** | 1,423 (11.9) |  |

NCEP-ATP III: National Cholesterol Education Program Adult Treatment Panel III; LDL-C: low-density lipoprotein cholesterol; LDL-C_F_: LDL-C calculated using the Friedewald formula; LDL-C_M-180_: LDL-C calculated using the original 180-cell Martin–Hopkins equation proposed by Martin et al [14]; LDL-C_KO-28_: LDL-C calculated using the 28-cell table (Fig 2) with the optimal ratios of triglycerides to very-low-density lipoprotein cholesterol (TG/VLDL-C) derived from our dataset.

*^a^* Values are presented as numbers (% within each LDL-C_F_ category). Boldface highlights cases in which the classification by LDL-C_M-180_ or LDL-C_KO-28_ differed from that of LDL-C_F_, based on directly measured LDL-C.

*^b^* Statistical significance of the differences in concordance between each LDL-C estimate and LDL-C_F_ was assessed using McNemar’s exact test for correlated proportions.

**(A)** **Reclassification using LDL-C_M-180_ by TG levels (B) Reclassification using LDL-C_KO-28_ by TG levels**

**(C) Reclassification using LDL-C_M-180_ by LDL-C_F_ levels (D) Reclassification using LDL-C_KO-28_ by LDL-C_F_ levels**

**in individuals with TG levels < 150 mg/dL in individuals with TG levels < 150 mg/dL**

**(E) Reclassification using LDL-C_M-180_ by LDL-C_F_ levels (F) Reclassification using LDL-C_KO-28_ by LDL-C_F_ levels**

**in individuals with TG levels of 150-399 mg/dL in individuals with TG levels of 150-399 mg/dL**

**Fig 7. Correct and incorrect reclassification using LDL-C_M-180_ and LDL-C_KO-28_ compared with LDL-C_F_.** Reclassification was defined as a shift in the NCEP-ATP III guideline-based treatment category initially assigned by LDL-C_F_ when using either LDL-C_M-180_ or LDL-C_KO-28_. The correctness of reclassification was evaluated against directly measured LDL-C, and each panel illustrates the proportions of correct (blue) and incorrect (gray) reclassifications. Panel (A) and Panel (B) show results across triglyceride categories (< 50, 50–99, 100–149, 150–199, 200–299, and 300–399) for LDL-C_M-180_ and LDL-C_KO-28_, respectively. Panel (C) and (D) present results among individuals with TG levels <150 mg/dL, stratified by LDL-C_F_–assigned treatment categories, using LDL-C_M-180_ and LDL-C_KO-28_, respectively. Panel (E) and (F) present the same comparison for individuals with TG levels of 150–399 mg/dL. **Abbreviations:** NCEP-ATP III: National Cholesterol Education Program Adult Treatment Panel III; LDL-C: low-density lipoprotein cholesterol; LDL-C_F_: LDL-C calculated using the Friedewald formula; LDL-C_M-180_: LDL-C calculated using the 180-cell Martin–Hopkins method proposed by Martin et al. [14]; LDL-C_KO-28_: LDL-C calculated using the 28-cell table (Fig 2) with the optimal ratios of triglycerides to very-low-density lipoprotein cholesterol (TG/VLDL-C) derived from our dataset.

**S13 Table. Reclassification outcomes using LDL-C_M-180_ and LDL-C_KO-28_ across treatment categories assigned by LDL-C_F_, as evaluated against directly measured LDL-C in individuals with TG levels < 150 mg/dL.**

|  |  | Reclassification using LDL-C_M-180_ *^a^* | | | Reclassification using LDL-C_KO-28_ *^a^* | | |
| --- | --- | --- | --- | --- | --- | --- | --- |
| LDL-C_F_, mg/dL | Classification | Correct | Incorrect | *p*-value *^b^* | Correct | Incorrect | *p*-value *^b^* |
| < 70 | Correct | 533 (80.4) | **24 ( 3.6)** | 0.077 | 535 (80.7) | **22 ( 3.3)** | 0.040 |
| (*n* = 663) | Incorrect | **39 ( 5.9)** | 67 (10.1) |  | **39 ( 5.9)** | 67 (10.1) |  |
| 70–99 | Correct | 2,506 (83.7) | **59 ( 2.0)** | < 0.001 | 2,504 (83.6) | **61 ( 2.0)** | < 0.001 |
| (*n* = 2,994) | Incorrect | **110 ( 3.7)** | 319 (10.7) |  | **135 ( 4.5)** | 294 ( 9.8) |  |
| 100–129 | Correct | 2,561 (81.2) | **89 ( 2.8)** | 0.051 | 2,592 (82.2) | **58 ( 1.8)** | 0.003 |
| (*n* = 3,153) | Incorrect | **118 ( 3.7)** | 385 (12.2) |  | **96 ( 3.0)** | 407 (12.9) |  |
| 130–159 | Correct | 1,196 (77.7) | **30 ( 1.9)** | < 0.001 | 1,167 (75.8) | **59 ( 3.8)** | < 0.001 |
| (*n* = 1,540) | Incorrect | **63 ( 4.1)** | 251 (16.3) |  | **107 ( 6.9)** | 207 (13.4) |  |
| 160–189 | Correct | 343 (74.9) | **8 ( 1.7)** | 0.076 | 331 (72.3) | **20 ( 4.4)** | 0.203 |
| (*n* = 458) | Incorrect | **18 ( 3.9)** | 89 (19.4) |  | **30 ( 6.6)** | 77 (16.8) |  |
| ≥ 190 | Correct | 74 (71.2) | **1 ( 1.0)** | 0.021 | 66 (63.5) | **9 ( 8.7)** | 1.000 |
| (*n* = 104) | Incorrect | **9 ( 8.7)** | 20 (19.2) |  | **9 ( 8.7)** | 20 (19.2) |  |
| Overall | Correct | 7,213 (80.9) | **211 ( 2.4)** | < 0.001 | 7,195 (80.7) | **229 ( 2.6)** | < 0.001 |
| (*n* = 8,912) | Incorrect | **357 ( 4.0)** | 1,131(12.7) |  | **416 ( 4.7)** | 1,072 (12.0) |  |

NCEP-ATP III: National Cholesterol Education Program Adult Treatment Panel III; LDL-C: low-density lipoprotein cholesterol; LDL-C_F_: LDL-C calculated using the Friedewald formula; LDL-C_M-180_: LDL-C calculated using the original 180-cell Martin–Hopkins equation proposed by Martin et al [14]; LDL-C_KO-28_: LDL-C calculated using the 28-cell table (Fig 2) with the optimal ratios of triglycerides to very-low-density lipoprotein cholesterol (TG/VLDL-C) derived from our dataset.

*^a^* Values are presented as numbers (% within each LDL-C_F_ category). Boldface highlights cases in which the classification by LDL-C_M-180_ or LDL-C_KO-28_ differed from that of LDL-C_F_, based on directly measured LDL-C.

*^b^* Statistical significance of the differences in concordance between each LDL-C estimate and LDL-C_F_ was assessed using McNemar’s exact test for correlated proportions.

**S14 Table. Reclassification outcomes using LDL-C_M-180_ and LDL-C_KO-28_ across treatment categories assigned by LDL-C_F_, as evaluated against directly measured LDL-C in individuals with TG levels of 150-399 mg/dL.**

|  |  | Reclassification using LDL-C_M-180_ *^a^* | | | Reclassification using LDL-C_KO-28_ *^a^* | | |
| --- | --- | --- | --- | --- | --- | --- | --- |
| LDL-C_F_, mg/dL | Classification | Correct | Incorrect | *p*-value *^b^* | Correct | Incorrect | *p*-value *^b^* |
| < 70 | Correct | 102 (33.9) | **41 (13.6)** | < 0.001 | 108 (35.9) | **35 (11.6)** | < 0.001 |
| (*n* = 301) | Incorrect | **147 (48.8)** | 11 ( 3.7) |  | **150 (49.8)** | 8 ( 2.7) |  |
| 70–99 | Correct | 355 (44.4) | **129 (16.1)** | < 0.001 | 382 (47.8) | **102 (12.8)** | < 0.001 |
| (*n* = 799) | Incorrect | **249 (32.1)** | 66 ( 8.3) |  | **243 (30.4)** | 72 ( 9.0) |  |
| 100–129 | Correct | 625 (62.2) | **96 ( 9.6)** | < 0.001 | 643 (64.0) | **78 ( 7.8)** | < 0.001 |
| (*n* = 1,005) | Incorrect | **150 (14.9)** | 134 (13.3) |  | **147 (14.6)** | 137 (13.6) |  |
| 130–159 | Correct | 468 (74.5) | **34 ( 5.4)** | 0.905 | 481 (76.6) | **21 ( 3.3)** | 0.169 |
| (*n* = 628) | Incorrect | **36 ( 5.7)** | 90 (14.3) |  | **32 ( 5.1)** | 94 (15.0) |  |
| 160–189 | Correct | 164 (74.9) | **6 ( 2.7)** | 0.508 | 150 (68.5) | **20 ( 9.1)** | 0.871 |
| (*n* = 219) | Incorrect | **3 ( 1.4)** | 46 (21.0) |  | **18 ( 8.2)** | 31 (14.2) |  |
| ≥ 190 | Correct | 56 (84.8) | **0 ( 0.0)** | 1.000 | 54 (81.8) | 2 ( 3.0) | 1.000 |
| (*n* = 66) | Incorrect | **0 ( 0.0)** | 10 (15.2) |  | **1 ( 1.5)** | 9 (13.6) |  |
| Overall | Correct | 1,770 (58.6) | **306 (10.1)** | < 0.001 | 1,818 (60.2) | **258 ( 8.5)** | < 0.001 |
| (*n* = 3,018) | Incorrect | **585 (19.4)** | 357 (11.8) |  | **591 (19.6)** | 351 (11.6) |  |

NCEP-ATP III: National Cholesterol Education Program Adult Treatment Panel III; LDL-C: low-density lipoprotein cholesterol; LDL-C_F_: LDL-C calculated using the Friedewald formula; LDL-C_M-180_: LDL-C calculated using the original 180-cell Martin–Hopkins equation proposed by Martin et al [14]; LDL-C_KO-28_: LDL-C calculated using the 28-cell table (Fig 2) with the optimal ratios of triglycerides to very-low-density lipoprotein cholesterol (TG/VLDL-C) derived from our dataset.

*^a^* Values are presented as numbers (% within each LDL-C_F_ category). Boldface highlights cases in which the classification by LDL-C_M-180_ or LDL-C_KO-28_ differed from that of LDL-C_F_, based on directly measured LDL-C.

*^b^* Statistical significance of the differences in concordance between each LDL-C estimate and LDL-C_F_ was assessed using McNemar’s exact test for correlated proportions.

**Point 3**: While tailoring TG/VLDL-C ratios to specific populations improves estimation accuracy, how feasible is it to implement this approach in clinical practice? Could the authors discuss practical implementation strategies—such as whether hospitals and laboratories can use population-specific median tables or if periodic recalibration would be necessary to maintain accuracy across different settings?

**Response 3**:

We fully agree that the clinical feasibility of implementation is an essential consideration. In response, we have revised the Discussion to include a dedicated paragraph addressing the feasibility, scalability, and operational considerations of our approach in real-world settings.

We acknowledge that the success of any estimation method hinges not only on its statistical accuracy but also on its adaptability in routine practice. To this end, we emphasize that many clinical laboratories already collect the full lipid profile—ncluding total cholesterol, HDL-cholesterol, and triglyceride (TG) concentrations—making the derivation and use of population-specific TG/VLDL-C ratio tables both feasible and low-cost.

Furthermore, we outline two major strategies:

1. **Local optimization**: Institutions with sufficient data infrastructure may generate TG/VLDL-C ratio tables tailored to their patient population and lipid assay platform.
2. **Validated reference table**: In settings where local recalibration is not feasible, hospitals may adopt reference tables derived from demographically and methodologically similar populations, with caution regarding assay compatibility.

We also recommend **periodic recalibration**, especially when switching assay platforms or observing shifts in population lipid profiles, as part of routine quality control procedures to maintain long-term accuracy.

This revision is reflected in the **Discussion** section on page 43–44 as follows:

**Practical implementation and scalability**

Many laboratories routinely collect lipid profile data, making local optimization of TG/VLDL-C ratios both feasible and potentially impactful. The appropriate number of stratification cells should be determined by the size and robustness of the available dataset. Our findings demonstrate that even a simplified 6-cell model based solely on TG levels (LDL-C_KO-6-TG_) can substantially improve concordance over LDL-C_F_, particularly among individuals with TG levels of 150-399 mg/dL (Fig 3B). When sufficient data are available, incorporating stratification by non–HDL-C to form a 12-cell model (LDL-C_KO-12_) can enhance performance.

However, increasing the number of strata should be approached with caution. As the number of cells or strata increases, the stability and reliability of the derived TG/VLDL-C ratios may decline, and the resulting performance gains may be only marginal. In settings where local recalibration is not feasible, validated ratio tables from demographically or methodologically similar populations may serve as a practical alternative. Nonetheless, as assay platforms and lipid distributions evolve over time, periodic reassessment and updating of TG/VLDL-C ratios should be integrated into routine quality control practices, particularly when transitioning to new laboratory assays.

We hope this revision provides a clear and actionable discussion of how our method can be adapted to a variety of clinical environments, and addresses the reviewer’s concerns about real-world applicability.

**Point 4**: Some parts of the text repeat the same points in different sections—for example, the limitations of the Friedewald formula in people with high triglycerides come up multiple times. It would help to streamline the writing by removing repetition and combining related results into a single summary table or figure for better clarity.

**Response 4**:

We appreciate the reviewer’s thoughtful comment. In response, we carefully reviewed the manuscript for redundant statements, particularly those related to the well-documented limitations of the Friedewald formula in individuals with elevated triglyceride (TG) levels.

To reduce repetition and improve logical flow, we revised multiple sections of the text for conciseness and removed overlapping content. Most notably, in the Discussion section, we eliminated the previous subsection titled “Limitations of the Friedewald formula”, which reiterated points already addressed in the Introduction and Results. Instead, we created a new, integrated subsection titled “Two major approaches for LDL-C estimation”, which concisely summarizes the limitations of the Friedewald formula and transitions into a broader conceptual discussion of the two main methodological alternatives that have emerged to overcome these limitations.

This restructuring allowed us to present the key points in a more cohesive and reader-friendly manner. The revised section not only avoids redundancy but also enhances the conceptual clarity of the manuscript by placing the Friedewald formula within the broader context of contemporary LDL-C estimation strategies. Moreover, It provides a clear rationale for the methodological focus of our study and acknowledges alternative approaches, thereby enriching the discussion.

This revision is reflected in the **Discussion** on pages 33–34 as follows:

**Two major approaches for LDL-C estimation**

Accurate assessment of LDL-C is of broad clinical interest and remains essential for evidence-based prevention of CVDs. Although beta quantification is the reference method, its high cost and technical complexity make it impractical for routine use. The Friedewald formula—based on a fixed TG/VLDL-C ratio of 5—has long served as a convenient alternative but is known to underestimate LDL-C in individuals with elevated TG and low LDL-C concentrations, which is consistent with our findings (Fig 6E).

To address the limitations of the Friedewald formula, several alternative equations have been developed over the years. Building on Friedewald’s concept of estimating LDL-C from the lipid profile, two major methodological approaches have emerged in the literature. One approach, exemplified by the Martin–Hopkins equation [14], utilizes strata-specific TG/VLDL-C ratios that vary across lipid ranges [17, 21]. The other approach, represented by the Sampson equation [15], employs multiple least squares regression to predict LDL-C using lipid parameters as independent variables [16, 18, 22–29].

An inherent limitation of the stratification-based approach is that increasing the number of strata requires a sufficiently large sample size to ensure stable and reliable ratio estimates, thereby restricting the number of covariates that can be incorporated when defining strata. In contrast, regression-based methods allow for the simultaneous inclusion of multiple predictors, even in relatively small samples. However, a key drawback is that regression coefficients may be highly sensitive to the characteristics of the training data—particularly the distribution of TG levels—raising concerns about their generalizability.

We focused on the strata-specific TG/VLDL-C ratio approach, as extended in the Martin–Hopkins method by incorporating both TG and non–HDL-C levels for stratification, due to its conceptual continuity with the Friedewald formula and thus its greater likelihood of clinical adoption. To further refine the Martin–Hopkins method, we applied optimal TG/VLDL-C ratios within each stratum to maximize concordance. However, exploring regression-based LDL-C prediction, including non-linear or machine learning models, remains a valid and promising alternative.

**Response to Reviewer 4 Comments**

Your detailed and encouraging comments were instrumental in sharpening our focus on critical issues in the manuscript. We have carefully considered each suggestion and responded to them individually below.

**Point 1**: Methods
Rather than optimizing for concordance within each stratum, why not directly fit the linear equation to predict LDL-C? Is it due to a lack of sample size? Mentioning why or why not that is possible would be beneficial.

**Response 1**:

Thank you for this important suggestion. In response, we would like to clarify our rationale for selecting a stratification-based optimization approach instead of directly fitting a regression model to predict LDL-C.

Given the limitations of the Friedewald equation, two major methodological streams have emerged in the literature for improving LDL-C estimation. One approach, exemplified by the Martin–Hopkins equation, uses stratification based on variables such as triglyceride (TG) and non–HDL-Cholesterol (non–HDL-C) to derive strata-specific TG/VLDL-C ratios. The other, represented by the Sampson-National Institutes of Health (NIH) equation, applies multiple least squares regression to predict LDL-C levels, using total cholesterol (TC), HDL-cholesterol (HDL-C), TG, and other lipid parameters as independent variables.

In this study, we focused on the former approach due to its conceptual continuity with the Friedewald formula, which remains the current standard of clinical practice. This equation uses a fixed TG/VLDL-C ratio of 5 for LDL-C estimation—a concept that is long-established and deeply embedded in clinical workflows. The Martin–Hopkins method extends this foundation by allowing the TG/VLDL-C ratio to vary across lipid strata, thereby better capturing physiological variability. Because it shares the same conceptual basis as the Friedewald method while offering improved accuracy, the Martin–Hopkins method is likely to be more readily adoptable in clinical settings compared to regression-based alternatives.

However, one limitation of the stratification-based approach is that as the number of strata increases, a larger sample size is required to ensure stable ratio estimates. This constraint limits the number of covariates that can be used when defining strata. By contrast, regression-based methods offer the advantage of considering multiple predictors simultaneously, even with relatively smaller sample sizes. Nevertheless, a key drawback is that regression coefficients may be highly sensitive to the underlying characteristics of the training data, particularly the distribution of TG levels.

To illustrate this sensitivity, we derived two regression equations based on the Sampson model framework using different subsets of our Korean dataset:

- A model based on Population 1 (*n* = 12,205), which included individuals with TG levels ≥ 400 mg/dL and reflects a TG distribution similar to that of the general Korean population, yielded one set of regression coefficients:

$$\text{Equation 1: LDL-C}=\frac{\text{TC}}{0.979}-\frac{\text{HDL-C}}{0.915}-\left(\frac{\text{TG}}{14.28}+\frac{\text{TG}\times\text{Non-HDL-C}}{1770}-\frac{\text{TG}^{2}}{15621}\right)-5.65$$

- A model based on the combined Population 1 and Population 2 (*n* = 19,664), also including individuals with TG levels ≥ 400 mg/dL, resulted in different coefficients:

$$\text{Equation 2: LDL-C}=\frac{\text{TC}}{0.960}-\frac{\text{HDL-C}}{0.970}-\left(\frac{\text{TG}}{15.90}+\frac{\text{TG}\times\text{Non-HDL-C}}{1648}-\frac{\text{TG}^{2}}{16075}\right)-12.36$$

These differences highlight the sensitivity of regression models to TG distribution, which can affect their generalizability. In contrast, the Martin–Hopkins method—by stratifying across TG and non–HDL-C ranges—may offer greater robustness across diverse lipid profiles.

For these reasons, we concentrated on refining the Martin–Hopkins method in this study, applying optimal TG/VLDL-C ratios within each stratum to maximize concordance. Nonetheless, we acknowledge that regression modeling remains a valid and promising alternative. In fact, when we applied the two regression equations described above, the overall concordance with directly measured LDL-C values was 83.4% and 83.3%, respectively, among individuals with TG levels < 400 mg/dL—comparable to our stratification-based results.

These findings suggest that both approaches may offer similar performance, and further research is needed to determine which provides sufficient improvement over the Friedewald formula to justify a change in clinical practice. Exploring regression-based LDL-C prediction, including non-linear or machine learning models, is a valuable direction for future studies and will be pursued in subsequent work.

We believe that these additions clarify our rationale for choosing a stratification-based optimization approach, and the following sentences have been added to the **Discussion** section on pages 33–34, as shown below:

To address the limitations of the Friedewald formula, several alternative equations have been developed over the years. Building on Friedewald’s concept of estimating LDL-C from the lipid profile, two major methodological approaches have emerged in the literature. One approach, exemplified by the Martin–Hopkins equation [14], utilizes strata-specific TG/VLDL-C ratios that vary across lipid ranges [17, 21]. The other approach, represented by the Sampson equation [15], employs multiple least squares regression to predict LDL-C using lipid parameters as independent variables [16, 18, 22–29].

An inherent limitation of the stratification-based approach is that increasing the number of strata requires a sufficiently large sample size to ensure stable and reliable ratio estimates, thereby restricting the number of covariates that can be incorporated when defining strata. In contrast, regression-based methods allow for the simultaneous inclusion of multiple predictors, even in relatively small samples. However, a key drawback is that regression coefficients may be highly sensitive to the characteristics of the training data—particularly the distribution of TG levels—raising concerns about their generalizability.

We focused on the strata-specific TG/VLDL-C ratio approach, as extended in the Martin–Hopkins method by incorporating both TG and non–HDL-C levels for stratification, due to its conceptual continuity with the Friedewald formula and thus its greater likelihood of clinical adoption. To further refine the Martin–Hopkins method, we applied optimal TG/VLDL-C ratios within each stratum to maximize concordance. However, exploring regression-based LDL-C prediction, including non-linear or machine learning models, remains a valid and promising alternative.

**Point 2**: Results
- There should be error bars, confidence intervals, p-values, or some measure of statistical significance when comparing concordance
- It may be informative to see evaluation done for more than just concordance. The review by Samuel et al (2023) of 23 equations for LDL-C that showed the MH equation to be the best had a 3-stage testing scheme that included concordance, MAE, stratification across demographic groups, and, lastly, clinical classification

Ref: Samuel C, Park J, Sajja A, Michos ED, Blumenthal RS, Jones SR, Martin SS. Accuracy of 23 Equations for Estimating LDL Cholesterol in a Clinical Laboratory Database of 5,051,467 Patients. Glob Heart. 2023 Jun 19;18(1):36. doi: 10.5334/gh.1214. PMID: 37361322; PMCID: PMC10289049

**Response 2**:

Thank you for this valuable suggestion. We agree that presenting statistical indicators alongside concordance enhances interpretability and robustness. In response, we have incorporated the following revisions and additions into the manuscript.

1. **Statistical comparison of concordance with confidence intervals and *p*-values**

In line with the reviewer’s recommendation, we enhanced our reporting of concordance results in **Figure 3** and **Supplementary Figure S1** as follows:

• Bars representing concordance rates ± 95% confidence intervals, calculated using the Clopper–Pearson exact method.

• Statistical comparisons between LDL-C estimation methods using McNemar’s exact test for correlated proportions, with corresponding *p*-values indicated by superscript lettering above each bar (methods sharing the same letter are not significantly different at *α* = 0.05).

• These details are described in the **Materials and Methods** (Data analysis) and **Results** sections, and summarized in **Supplementary Tables S9**, **S10**, **S15**, and **S16**.

2. **Inclusion of model fit and error metrics**

Following the framework described by Samuel et al. (2023), we expanded our analysis to include model fit and error metrics, specifically:

• Coefficient of determination (*R*^2^)

• Mean absolute error (MAE)

• Mean squared error (MSE)

• Mean relative error (MRE)

These metrics were used to evaluate the accuracy of each LDL-C estimation method in comparison with directly measured LDL-C. Results are summarized in **S8 Table** and discussed in the **Materials and Methods** (Data analysis) and **Results** sections.

3. **Bland–Altman analysis for method agreement**

To further assess agreement with directly measured LDL-C (LDL-C_D_), we conducted Bland–Altman analyses for three LDL-C estimation methods: LDL-C_F_, LDL-C_M-180_, and LDL-C_KO-28_. These plots visualize the bias (difference between LDL-C_E_ and LDL-C_D_) against the mean of the two values and illustrate the extent of agreement. The mean bias and the 95% limits of agreement (mean ± 1.96 SD) were calculated to evaluate systematic differences and dispersion across the range of LDL-C values. The results are presented in **Figure 4** and described in the **Materials and Methods** (Data analysis) and **Results** sections of the revised manuscript.

These additional analyses provide a more comprehensive assessment of estimation accuracy, classification concordance, and potential clinical implications, as recommended. The revised figures and tables have been incorporated into the manuscript and are also appended to this response letter for your convenience.

**Changes Made:**

• **Materials and Methods – Data analysis** (pages 10–11): We added new paragraphs describing the Bland–Altman analysis and model fit/error metrics, and also revised a portion of the preceding paragraph to include McNemar’s exact test, Clopper–Pearson confidence intervals.

LDL-C values, including both directly measured (LDL-C_D_) and estimated LDL-C (LDL-C_E_), were classified according to the NCEP-ATP III guideline into six categories (< 70, 70–99, 100–129, 130–159, 160–189, and ≥ 190 mg/dL). The concordance rate was defined as the proportion of cases in which LDL-C_D_ fell into the same category as the corresponding LDL-C_E_, with initial classification based on the estimated values. Cross-tabulations were constructed to assess concordance between each LDL-C_E_ and LDL-C_D_. McNemar’s exact test for correlated proportions was used to compare concordance rates between different LDL-C estimation methods. The 95% confidence intervals for concordance rates were calculated using the Clopper–Pearson exact method.

Bland–Altman analysis was performed to assess the agreement between LDL-C_E_ and LDL-C_D_. For three estimation methods (LDL-C_F_, LDL-C_M-180_, and LDL-C_KO-28_), the bias (difference between LDL-C_E_ and LDL-C_D_) was plotted against the mean of the two values. The mean bias and the 95% limits of agreement (mean ± 1.96 SD) were calculated to evaluate systematic differences and dispersion across the range of LDL-C values. In addition, model fit and error metrics were assessed for each LDL-C estimation method in comparison with LDL-C_D_, including the coefficient of determination (*R*^2^), mean relative error (MRE), mean absolute error (MAE), and mean squared error (MSE).

• **Results – Figure 3** (pages 21–23): We presented the concordance results with detailed statistical comparisons between LDL-C estimation methods. These include 95% confidence intervals for concordance rates (calculated using the Clopper–Pearson exact method) and *p*-values from McNemar’s exact test.

Fig 3 illustrates the concordance rates of LDL-C estimates calculated using the Friedewald and Martin–Hopkins methods, compared with directly measured LDL-C, based on the NCEP-ATP III guideline classification. The results are stratified by TG levels, with Fig 3A showing concordance for TG levels < 150 mg/dL and Fig 3B for TG levels of 150–399 mg/dL. LDL-C estimates based on the Martin–Hopkins method included not only LDL-C_M-180_, calculated using the original 180-cell Martin–Hopkins equation, but also LDL-C_KO-6_, LDL-C_KO-12_, and LDL-C_KO-28_, calculated using the optimal TG/VLDL-C ratios derived from our dataset. The statistical significance of differences in concordance between these LDL-C estimates is detailed in S9 and S10 Tables.

Among individuals with TG levels < 150 mg/dL (Fig 3A), all four Martin–Hopkins-based estimates showed statistically significantly higher concordance than the Friedewald-calculated LDL-C (LDL-C_F_); however, the magnitude of improvement was modest, with increases of approximately 2%. In contrast, among individuals with TG levels of 150–399 mg/dL (Fig 3B), each of the Martin–Hopkins-based estimates demonstrated a substantial improvement over LDL-C_F_, with concordance higher by approximately 10% (*p* < 0.001 for all comparisons).

• **Results – Figure 3 legend** (page 22):

**Fig 3. Comparison of concordance rates between Friedewald and Martin–Hopkins LDL-C estimates by TG levels: < 150 mg/dL and 150–399 mg/dL.** Panel (A) shows results for individuals with TG levels < 150 mg/dL, and Panel (B) for those with TG levels of 150–399 mg/dL. Bars represent concordance rates ± 95% confidence intervals for each LDL-C estimate. From left to right, bars correspond to the following LDL-C estimates: LDL-C_F_, LDL-C_M-180_, LDL-C_KO-6_, LDL-C_KO-12_, and LDL-C_KO-28_. * Bars labeled with the same letters indicate no statistically significant difference in concordance between the corresponding LDL-C estimates (*α* = 0.05), based on pairwise comparisons using McNemar’s exact test for correlated proportions (see S9 and S10 Tables). The 95% confidence intervals for concordance rates were calculated using the Clopper–Pearson exact method. **Abbreviations:** NCEP-ATP III: National Cholesterol Education Program Adult Treatment Panel III; TG: triglyceride; LDL-C: low-density lipoprotein cholesterol; LDL-C_F_: LDL-C calculated using the Friedewald formula; LDL-C_M-180_: LDL-C calculated using the original 180-cell Martin–Hopkins equation proposed by Martin et al. [14]; LDL-C_KO-N_ (LDL-C_KO-6-TG_, LDL-C_KO-12_, and LDL-C_KO-28_): LDL-C calculated using the N-cell tables with the optimal ratios of triglycerides to very-low-density lipoprotein cholesterol (TG/VLDL-C) derived from our dataset.

• **Results – Supplementary Figure S1** (page 31): We conducted a subgroup analysis to assess the impact of concordance differences on classification outcomes among individuals classified as having LDL-C < 70 mg/dL by the Fridewald formula.

Among 964 individuals classified as having LDL-C < 70 mg/dL by LDL-C_F_, S1 Fig presents the concordance rates of LDL-C estimates calculated using the Friedewald and Martin–Hopkins-based methods, compared with directly measured LDL-C. This analysis follows the NCEP-ATP III guideline and is stratified by TG levels: < 150 mg/dL (S1A Fig) and 150–399 mg/dL (S1B Fig). The Martin–Hopkins-based estimates included not only LDL-C_M-180_ but also LDL-C_KO-6_, LDL-C_KO-12_, and LDL-C_KO-28_, which were calculated using optimized TG/VLDL-C ratios derived from our dataset. The statistical significance of differences in overall concordance between these LDL-C estimates was assessed, as detailed in S15 and S16 Tables. Among individuals with TG levels < 150 mg/dL, all Martin–Hopkins-based LDL-estimates—except LDL-C_M-180_—showed statistically significant improvements in concordance compared to LDL-C_F_; however, the magnitude of improvement was modest, with increases of less than 3%. In contrast, for individuals with TG levels of 150–399 mg/dL, all four Martin–Hopkins-based estimates demonstrated substantial improvements in concordance compared to LDL-C_F_, with increases exceeding 30% (*p* < 0.001 for all comparisons).

• **Results – Supplementary Figure S1 legend** (pages 53):

**S1 Fig. Concordance rates of LDL-C estimates using Friedewald and Martin–Hopkins methods among individuals with LDL-C_F_ < 70 mg/dL, compared with directly measured LDL-C, according to the NCEP-ATP III guideline.** Bars represent concordance rates ± 95% confidence intervals for LDL-C estimates, stratified by triglyceride levels of < 150 mg/dL (Panel A) and 150–399 mg/dL (Panel B). From left to right, bars correspond to the following LDL-C estimates: LDL-C_F_, LDL-C_M-180_, LDL-C_KO-6_, LDL-C_KO-12_, and LDL-C_KO-28_. * Bars labeled with the same letters indicate that no statistically significant difference in concordance between LDL-C estimates (*α* = 0.05), based on pairwise comparisons using McNemar’s exact test for correlated proportions (see S15 and S16 Tables for details). The 95% confidence intervals for concordance rates were calculated using the Clopper–Pearson exact method. **Abbreviations:** NCEP-ATP III: National Cholesterol Education Program Adult Treatment Panel III; TG: triglyceride; LDL-C: low-density lipoprotein cholesterol; LDL-C_F_: LDL-C calculated using the Friedewald formula; LDL-C_M-180_: LDL-C calculated using the original 180-cell Martin–Hopkins equation proposed by Martin et al. [14]; LDL-C_KO-N_ (LDL-C_KO-6-TG_, LDL-C_KO-12_, and LDL-C_KO-28_): LDL-C calculated using the N-cell tables with the optimal ratios of triglycerides to very-low-density lipoprotein cholesterol (TG/VLDL-C) derived from our dataset.

• **Results – S8 Table** (page 21): We assessed model fit and error metrics for each LDL-C estimation method in comparison with LDL-C_D_, using *R*^2^, MRE, MAE, and MSE.

In addition to overall concordance rates, model fit and error metrics were evaluated for each LDL-C estimation method in comparison with directly measured LDL-C, as summarized in S8 Table. In this table, the methods are ranked by descending overall concordance. LDL-C_KO-28_ yielded the lowest mean absolute error (5.09 mg/dL), smallest mean squared error (47.64 mg/dL^2^), and lowest mean relative error (0.73%) while achieving the highest *R*^2^ value (0.954). The 180-cell Martin–Hopkins equation also demonstrated excellent performance, with a mean absolute error of 5.11 mg/dL and *R*^2^ of 0.952. These results support the superior estimation accuracy of our optimized method (LDL-C_KO-28_) over both the Friedewald formula and other alternatives.

• **Results – Figure 4** (pages 23–24): We described the Bland–Altman plots that illustrate bias and agreement across LDL-C estimation methods. This includes the mean bias and the 95% limits of agreement (mean ± 1.96 SD) for three estimation methods.

To further assess the agreement between estimated LDL-C (LDL-C_E_) and directly measured LDL-C (LDL-C_D_), Bland–Altman analyses were conducted for three estimation methods: LDL-C_F_, LDL-C_M-180_, and LDL-C_KO-28_. As shown in Fig 4, each panel displays the bias (i.e., the difference between LDL-C_E_ and LDL-C_D_) plotted against the mean of the two values. The black dashed line indicates the mean bias, while the orange and red dashed lines represent the upper and lower 95% limits of agreement (mean ± 1.96 SD), respectively. Compared to LDL-C_F_, both LDL-C_M-180_ and LDL-C_KO-28_ exhibited reduced bias dispersion and narrower limits of agreement, indicating improved concordance with LDL-C_D_ values. In particular, for individuals with TG levels of 300–399 mg/dL (represented by green dots), the mean bias and 95% limits of agreement (mean ± 1.96 SD) were: −13.49 ± 28.61 mg/dL for LDL-C_F_, 6.69 ± 26.19 mg/dL for LDL-C_M-180_, and 2.61 ± 25.51 mg/dL for LDL-C_KO-28_. These findings provide additional evidence supporting the improved accuracy of the optimized estimation method (LDL-C_KO-28_), especially among individuals with elevated TG levels.

• **Results – Figure 4 legend**: (page 24):

**Fig 4. Bland–Altman plots comparing estimated LDL-C values with directly measured LDL-C**. Each panel shows the bias (i.e., the difference between LDL-C_E_ and LDL-C_D_) plotted against the mean of LDL-C_D_ and LDL-C_E_. The black dashed line represents the mean bias, while the orange and red dashed lines indicate the upper and lower 95% limit of agreement (mean ± 1.96 SD), respectively. Panels (A), (B), and (C) correspond to LDL-C_F_, LDL-C_M-180_, and LDL-C_KO-28_, respectively. These plots illustrate differences in bias magnitude and dispersion, reflecting the degree of agreement between each LDL-C_E_ and LDL-C_D_. Green dots represent individuals with TG levels of 300–399 mg/dL. **Abbreviations:** LDL-C: low-density lipoprotein cholesterol; TG: triglyceride; LDL-C_E_: estimated LDL-C; LDL-C_D_: LDL-C directly measured using a homogeneous enzymatic assay; LDL-C_F_: LDL-C calculated using the Friedewald formula; LDL-C_M-180_: LDL-C calculated using the original 180-cell Martin–Hopkins equation proposed by Martin et al. [14]; LDL-C_KO-28_: LDL-C calculated using the 28-cell table (Fig 2) with the optimal ratios of TG/VLDL-C (triglycerides to very-low-density lipoprotein cholesterol) derived from our dataset; SD: standard deviation.

**(A) Concordance in individuals with TG < 150 mg/dL (B) Concordance in individuals with TG levels of 150-399 mg/dL**

**Fig 3. Comparison of concordance rates between Friedewald and Martin–Hopkins LDL-C estimates by TG levels: < 150 mg/dL and 150–399 mg/dL.** Panel (A) shows results for individuals with TG levels < 150 mg/dL, and Panel (B) for those with TG levels of 150–399 mg/dL. Bars represent concordance rates ± 95% confidence intervals for LDL-C estimates. From left to right, bars correspond to the following LDL-C estimates: LDL-C_F_, LDL-C_M-180_, LDL-C_KO-6_, LDL-C_KO-12_, and LDL-C_KO-28_. * Bars labeled with the same letters indicate that no statistically significant difference in concordance between LDL-C estimates (*α* = 0.05), based on pairwise comparisons using McNemar’s exact test for correlated proportions (see S9 and S10 Tables). The 95% confidence intervals for concordance rates were calculated using the Clopper–Pearson exact method. **Abbreviations:** NCEP-ATP III: National Cholesterol Education Program Adult Treatment Panel III; TG: triglyceride; LDL-C: low-density lipoprotein cholesterol; LDL-C_F_: LDL-C calculated using the Friedewald formula; LDL-C_M-180_: LDL-C calculated using the original 180-cell Martin–Hopkins equation proposed by Martin et al. [14]; LDL-C_KO-N_ (LDL-C_KO-6-TG_, LDL-C_KO-12_, and LDL-C_KO-28_): LDL-C calculated using the N-cell tables with the optimal ratios of triglycerides to very-low-density lipoprotein cholesterol (TG/VLDL-C) derived from our dataset.

**S9 Table. Statistical significance of differences in overall concordance between LDL-C estimates among individuals with triglyceride levels < 150 mg/dL.**

|  | Estimated LDL-C (LDL-C_E_) *^a^* | | | | | | | | | | | | |
| --- | --- | --- | --- | --- | --- | --- | --- | --- | --- | --- | --- | --- | --- |
| LDL-C_E_ | M-10 | M-180 | KM-6-TG | KO-6-TG | KM-10 | KO-10 | KM-12-TG | KO-12-TG | KM-12 | KO-12 | KM-28 | KO-28 | KM-180 |
| LDL-C_F_ | <0.001 | <0.001 | <0.001 | <0.001 | 0.001 | <0.001 | <0.001 | <0.001 | <0.001 | <0.001 | <0.001 | <0.001 | <0.001 |
| LDL-C_M-10_ | NA | 0.021 | 0.703 | 0.075 | 0.569 | 0.114 | 0.211 | 0.011 | 0.45 | <0.001 | 0.106 | <0.001 | 0.004 |
| LDL-C_M-180_ | 0.021 | NA | 0.044 | 0.465 | 0.01 | 0.354 | 0.244 | 0.7 | 0.635 | 0.296 | 0.342 | 0.048 | 0.211 |
| LDL-C_KM-6-TG_ *^b^* | 0.703 | 0.044 | NA | 0.069 | 0.39 | 0.444 | 0.226 | 0.012 | 0.072 | 0.004 | 0.192 | <0.001 | 0.004 |
| LDL-C_KO-6-TG_ *^b^* | 0.075 | 0.465 | 0.069 | NA | 0.069 | 0.787 | 0.757 | 0.193 | 0.835 | 0.063 | 0.794 | 0.008 | 0.085 |
| LDL-C_KM-10_ | 0.569 | 0.01 | 0.39 | 0.069 | NA | 0.04 | 0.103 | 0.006 | 0.008 | <0.001 | 0.047 | <0.001 | 0.001 |
| LDL-C_KO-10_ | 0.114 | 0.354 | 0.444 | 0.787 | 0.04 | NA | 1 | 0.212 | 0.568 | 0.003 | 0.961 | <0.001 | 0.094 |
| LDL-C_KM-12-TG_ *^b^* | 0.211 | 0.244 | 0.226 | 0.757 | 0.103 | 1 | NA | 0.121 | 0.646 | 0.065 | 0.006 | 0.01 | 0.038 |
| LDL-C_KO-12-TG_ *^b^* | 0.011 | 0.7 | 0.012 | 0.193 | 0.006 | 0.212 | 0.121 | NA | 0.401 | 0.534 | 0.208 | 0.126 | 0.62 |
| LDL-C_KM-12_ | 0.45 | 0.635 | 0.072 | 0.835 | 0.008 | 0.568 | 0.646 | 0.401 | NA | 0.062 | 0.498 | 0.004 | 0.142 |
| LDL-C_KO-12_ | <0.001 | 0.296 | 0.004 | 0.063 | <0.001 | 0.003 | 0.065 | 0.534 | 0.062 | NA | 0.039 | 0.142 | 0.961 |
| LDL-C_KM-28_ | 0.106 | 0.342 | 0.192 | 0.794 | 0.047 | 0.961 | 0.006 | 0.208 | 0.498 | 0.039 | NA | 0.001 | 0.041 |
| LDL-C_KO-28_ | <0.001 | 0.048 | <0.001 | 0.008 | <0.001 | <0.001 | 0.01 | 0.126 | 0.004 | 0.142 | 0.001 | NA | 0.001 |
| LDL-C_KM-180_ | 0.004 | 0.211 | 0.004 | 0.085 | 0.001 | 0.094 | 0.038 | 0.62 | 0.142 | 0.961 | 0.041 | 0.001 | NA |

LDL-C: low-density lipoprotein cholesterol; LDL-C_E_: estimated LDL-C; LDL-C_F_: LDL-C calculated using the Friedewald formula; LDL-C_M-N_ (LDL-C_M-10_ and LDL-C_M-180_): LDL-C calculated using the N-cell Martin–Hopkins method described by Martin et al [14]; TG/VLDL-C ratio: ratio of triglycerides to very-low-density lipoprotein cholesterol; LDL-C_KM-N_ (LDL-C_KM-6-TG_, LDL-C_KM-10_, LDL-C_KM-12-TG_, LDL-C_KM-12_, LDL-C_KM-28_, and LDL-C_KM-180_): LDL-C calculated using the N-cell tables with the median TG/VLDL-C ratios derived from our dataset; LDL-C_KO-N_ (LDL-C_KO-6-TG_, LDL-C_KO-10_, LDL-C_KO-12-TG_, LDL-C_KO-12_, and LDL-C_KO-28_): LDL-C calculated using the N-cell tables with the optimal TG/VLDL-C ratios derived from our dataset; NA, not applicable.

*^a^* The values in the table are *p*-values. Statistical significance of differences in overall concordance between two LDL-C estimates was assessed using McNemar’s exact test for correlated proportions.

*^b^* When stratification was based on TG levels alone, rather than combined TG and non–HDL-C levels, the subscript “_TG_” was added, as in LDL-C_KM-N-TG_ or LDL-C_KO-N-TG_.

**S10 Table. Statistical significance of differences in overall concordance between LDL-C estimates among individuals with triglyceride levels of 150–399 mg/dL.**

|  | Estimated LDL-C (LDL-C_E_) *^a^* | | | | | | | | | | | | |
| --- | --- | --- | --- | --- | --- | --- | --- | --- | --- | --- | --- | --- | --- |
| LDL-C_E_ | M-10 | M-180 | KM-6-TG | KO-6-TG | KM-10 | KO-10 | KM-12-TG | KO-12-TG | KM-12 | KO-12 | KM-28 | KO-28 | KM-180 |
| LDL-C_F_ | <0.001 | <0.001 | <0.001 | <0.001 | <0.001 | <0.001 | <0.001 | <0.001 | <0.001 | <0.001 | <0.001 | <0.001 | <0.001 |
| LDL-C_M-10_ | NA | 0.89 | 0.253 | 0.214 | 0.395 | 0.02 | 0.253 | 0.109 | 0.176 | 0.004 | 0.001 | <0.001 | 0.002 |
| LDL-C_M-180_ | 0.89 | NA | 0.449 | 0.443 | 0.687 | 0.128 | 0.449 | 0.223 | 0.45 | 0.034 | 0.002 | <0.001 | 0.003 |
| LDL-C_KM-6-TG_ *^b^* | 0.253 | 0.449 | NA | 1 | 0.681 | 0.353 | 1 | 0.512 | 1 | 0.066 | 0.041 | 0.008 | 0.048 |
| LDL-C_KO-6-TG_ *^b^* | 0.214 | 0.443 | 1 | NA | 1 | 0.275 | 1 | 0.516 | 1 | 0.095 | 0.044 | 0.008 | 0.049 |
| LDL-C_KM-10_ | 0.395 | 0.687 | 0.681 | 1 | NA | 0.198 | 0.681 | 0.341 | 0.542 | 0.008 | 0.004 | 0.001 | 0.012 |
| LDL-C_KO-10_ | 0.02 | 0.128 | 0.353 | 0.275 | 0.198 | NA | 0.353 | 0.92 | 0.379 | 0.416 | 0.13 | 0.026 | 0.195 |
| LDL-C_KM-12-TG_ *^b^* | 0.253 | 0.449 | 1 | 1 | 0.681 | 0.353 | NA | 0.512 | 1 | 0.066 | 0.028 | 0.008 | 0.048 |
| LDL-C_KO-12-TG_ *^b^* | 0.109 | 0.223 | 0.512 | 0.516 | 0.341 | 0.92 | 0.512 | NA | 0.55 | 0.419 | 0.155 | 0.039 | 0.194 |
| LDL-C_KM-12_ | 0.176 | 0.45 | 1 | 1 | 0.542 | 0.379 | 1 | 0.55 | NA | 0.025 | 0.007 | 0.003 | 0.026 |
| LDL-C_KO-12_ | 0.004 | 0.034 | 0.066 | 0.095 | 0.008 | 0.416 | 0.066 | 0.419 | 0.025 | NA | 0.344 | 0.081 | 0.455 |
| LDL-C_KM-28_ | 0.001 | 0.002 | 0.041 | 0.044 | 0.004 | 0.13 | 0.028 | 0.155 | 0.007 | 0.344 | NA | 0.363 | 0.92 |
| LDL-C_KO-28_ | <0.001 | <0.001 | 0.008 | 0.008 | 0.001 | 0.026 | 0.008 | 0.039 | 0.003 | 0.081 | 0.363 | NA | 0.363 |
| LDL-C_KM-180_ | 0.002 | 0.003 | 0.048 | 0.049 | 0.012 | 0.195 | 0.048 | 0.194 | 0.026 | 0.455 | 0.92 | 0.363 | NA |

LDL-C: low-density lipoprotein cholesterol; LDL-C_E_: estimated LDL-C; LDL-C_F_: LDL-C calculated using the Friedewald formula; LDL-C_M-N_ (LDL-C_M-10_ and LDL-C_M-180_): LDL-C calculated using the N-cell Martin–Hopkins method described by Martin et al [14]; TG/VLDL-C ratio: ratio of triglycerides to very-low-density lipoprotein cholesterol; LDL-C_KM-N_ (LDL-C_KM-6-TG_, LDL-C_KM-10_, LDL-C_KM-12-TG_, LDL-C_KM-12_, LDL-C_KM-28_, and LDL-C_KM-180_): LDL-C calculated using the N-cell tables with the median TG/VLDL-C ratios derived from our dataset; LDL-C_KO-N_ (LDL-C_KO-6-TG_, LDL-C_KO-10_, LDL-C_KO-12-TG_, LDL-C_KO-12_, and LDL-C_KO-28_): LDL-C calculated using the N-cell tables with the optimal TG/VLDL-C ratios derived from our dataset; NA, not applicable.

*^a^* The values in the table are *p*-values. Statistical significance of differences in overall concordance between two LDL-C estimates was assessed using McNemar’s exact test for correlated proportions.

*^b^* When stratification was based on TG levels alone, rather than combined TG and non–HDL-C levels, the subscript “_TG_” was added, as in LDL-C_KM-N-TG_ or LDL-C_KO-N-TG_.

**(A) Concordance in individuals with TG < 150 mg/dL (B) Concordance in individuals with TG levels of 150-399 mg/dL**

**S1 Fig. Concordance rates of LDL-C estimates using Friedewald and Martin–Hopkins methods among individuals with LDL-C_F_ < 70 mg/dL, compared with directly measured LDL-C, according to the NCEP-ATP III guideline.** Bars represent concordance rates ± 95% confidence intervals for LDL-C estimates, stratified by triglyceride levels of < 150 mg/dL (Panel A) and 150–399 mg/dL (Panel B). From left to right, bars correspond to the following LDL-C estimates: LDL-C_F_, LDL-C_M-180_, LDL-C_KO-6_, LDL-C_KO-12_, and LDL-C_KO-28_. * Bars labeled with the same letters indicate that no statistically significant difference in concordance between LDL-C estimates (*α* = 0.05), based on pairwise comparisons using McNemar’s exact test for correlated proportions (see S15 and S16 Tables for details). The 95% confidence intervals for concordance rates were calculated using the Clopper–Pearson exact method. **Abbreviations:** NCEP-ATP III: National Cholesterol Education Program Adult Treatment Panel III; TG: triglyceride; LDL-C: low-density lipoprotein cholesterol; LDL-C_F_: LDL-C calculated using the Friedewald formula; LDL-C_M-180_: LDL-C calculated using the original 180-cell Martin–Hopkins equation proposed by Martin et al. [14]; LDL-C_KO-N_ (LDL-C_KO-6-TG_, LDL-C_KO-12_, and LDL-C_KO-28_): LDL-C calculated using the N-cell tables with the optimal ratios of triglycerides to very-low-density lipoprotein cholesterol (TG/VLDL-C) derived from our dataset.

**S15 Table. Statistical significance of differences in overall concordance between LDL-C estimates among individuals with triglyceride levels < 150 mg/dL and LDL-C_F_ < 70mg/dL.**

|  | Estimated LDL-C (LDL-C_E_) *^a^* | | | | | | | | | | | | |
| --- | --- | --- | --- | --- | --- | --- | --- | --- | --- | --- | --- | --- | --- |
| LDL-C_E_ | M-10 | M-180 | KM-6-TG | KO-6-TG | KM-10 | KO-10 | KM-12-TG | KO-12-TG | KM-12 | KO-12 | KM-28 | KO-28 | KM-180 |
| LDL-C_F_ | 0.028 | 0.077 | 0.011 | 0.028 | 0.072 | 0.04 | 0.011 | 0.009 | 0.065 | 0.04 | 0.065 | 0.04 | 0.033 |
| LDL-C_M-10_ | NA | 1 | 1 | 1 | 0.625 | 0.824 | 1 | 0.267 | 1 | 0.824 | 1 | 0.824 | 0.678 |
| LDL-C_M-180_ | 0.077 | NA | 1 | 1 | 0.851 | 0.824 | 1 | 0.227 | 1 | 0.824 | 1 | 0.824 | 0.648 |
| LDL-C_KM-6-TG_ *^b^* | 0.011 | 1 | NA | 1 | 0.791 | 0.856 | 1 | 0.405 | 1 | 0.856 | 0.014 | 0.856 | 0.728 |
| LDL-C_KO-6-TG_ *^b^* | 0.028 | 1 | 1 | NA | 0.625 | 0.824 | 1 | 0.267 | 1 | 0.824 | 1 | 0.824 | 0.678 |
| LDL-C_KM-10_ | 0.072 | 0.625 | 0.851 | 0.791 | NA | 0.454 | 0.791 | 0.143 | 1 | 0.454 | 1 | 0.454 | 0.359 |
| LDL-C_KO-10_ | 0.04 | 0.824 | 0.824 | 0.856 | 0.824 | NA | 0.856 | 0.549 | 0.549 | 1 | 0.549 | 1 | 1 |
| LDL-C_KM-12-TG_ *^b^* | 0.011 | 1 | 1 | 1 | 1 | 0.791 | NA | 0.405 | 1 | 0.856 | 1 | 0.856 | 0.728 |
| LDL-C_KO-12-TG_ *^b^* | 0.009 | 0.267 | 0.227 | 0.405 | 0.267 | 0.143 | 0.549 | NA | 0.18 | 0.549 | 0.18 | 0.549 | 0.832 |
| LDL-C_KM-12_ | 0.065 | 1 | 1 | 1 | 1 | 1 | 0.549 | 1 | NA | 0.549 | 1 | 0.549 | 0.481 |
| LDL-C_KO-12_ | 0.04 | 0.824 | 0.824 | 0.856 | 0.824 | 0.454 | 1 | 0.856 | 0.549 | NA | 0.549 | 1 | 1 |
| LDL-C_KM-28_ | 0.065 | 1 | 1 | 0.014 | 1 | 1 | 0.549 | 1 | 0.18 | 1 | NA | 0.549 | 0.549 |
| LDL-C_KO-28_ | 0.04 | 0.824 | 0.824 | 0.856 | 0.824 | 0.454 | 1 | 0.856 | 0.549 | 0.549 | 1 | NA | 1 |
| LDL-C_KM-180_ | 0.033 | 0.678 | 0.648 | 0.728 | 0.678 | 0.359 | 1 | 0.728 | 0.832 | 0.481 | 1 | 0.549 | NA |

LDL-C: low-density lipoprotein cholesterol; LDL-C_E_: estimated LDL-C; LDL-C_F_: LDL-C calculated using the Friedewald formula; LDL-C_M-N_ (LDL-C_M-10_ and LDL-C_M-180_): LDL-C calculated using the N-cell Martin–Hopkins method described by Martin et al [14]; TG/VLDL-C ratio: ratio of triglycerides to very-low-density lipoprotein cholesterol; LDL-C_KM-N_ (LDL-C_KM-6-TG_, LDL-C_KM-10_, LDL-C_KM-12-TG_, LDL-C_KM-12_, LDL-C_KM-28_, and LDL-C_KM-180_): LDL-C calculated using the N-cell tables with the median TG/VLDL-C ratios derived from our dataset; LDL-C_KO-N_ (LDL-C_KO-6-TG_, LDL-C_KO-10_, LDL-C_KO-12-TG_, LDL-C_KO-12_, and LDL-C_KO-28_): LDL-C calculated using the N-cell tables with the optimal TG/VLDL-C ratios derived from our dataset; NA, not applicable.

*^a^* The values in the table are *p*-values. Statistical significance of differences in overall concordance between two LDL-C estimates was assessed using McNemar’s exact test for correlated proportions.

*^b^* When stratification was based on TG levels alone, rather than combined TG and non–HDL-C levels, the subscript “_TG_” was added, as in LDL-C_KM-N-TG_ or LDL-C_KO-N-TG_.

**S16 Table. Statistical significance of differences in overall concordance between LDL-C estimates among individuals with triglyceride levels of 150–399 mg/dL and LDL-C_F_ < 70mg/dL.**

|  | Estimated LDL-C (LDL-C_E_) *^a^* | | | | | | | | | | | | |
| --- | --- | --- | --- | --- | --- | --- | --- | --- | --- | --- | --- | --- | --- |
| LDL-C_E_ | M-10 | M-180 | KM-6-TG | KO-6-TG | KM-10 | KO-10 | KM-12-TG | KO-12-TG | KM-12 | KO-12 | KM-28 | KO-28 | KM-180 |
| LDL-C_F_ | <0.001 | <0.001 | <0.001 | <0.001 | <0.001 | <0.001 | <0.001 | <0.001 | <0.001 | <0.001 | <0.001 | <0.001 | <0.001 |
| LDL-C_M-10_ | NA | 0.791 | 0.659 | 0.878 | 0.791 | 0.152 | 0.659 | 0.511 | 0.581 | 0.035 | 0.454 | 0.08 | 0.824 |
| LDL-C_M-180_ | <0.001 | NA | 0.519 | 0.689 | 0.572 | 0.418 | 0.519 | 0.775 | 1 | 0.248 | 0.832 | 0.108 | 0.572 |
| LDL-C_KM-6-TG_ *^b^* | <0.001 | 0.659 | NA | 0.625 | 0.864 | 0.017 | 1 | 0.022 | 0.296 | 0.035 | 0.442 | 0.063 | 0.864 |
| LDL-C_KO-6-TG_ *^b^* | <0.001 | 0.878 | 0.689 | NA | 1 | 0.031 | 0.625 | 0.092 | 0.458 | 0.061 | 0.405 | 0.098 | 1 |
| LDL-C_KM-10_ | <0.001 | 0.791 | 0.572 | 0.864 | NA | 0.006 | 0.864 | 0.23 | 0.063 | 0.007 | 0.146 | 0.047 | 1 |
| LDL-C_KO-10_ | <0.001 | 0.152 | 0.418 | 0.017 | 0.031 | NA | 0.017 | 0.581 | 0.227 | 1 | 0.481 | 0.736 | 0.031 |
| LDL-C_KM-12-TG_ *^b^* | <0.001 | 0.659 | 0.519 | 1 | 0.625 | 0.864 | NA | 0.022 | 0.296 | 0.035 | 0.268 | 0.063 | 0.864 |
| LDL-C_KO-12-TG_ *^b^* | <0.001 | 0.511 | 0.775 | 0.022 | 0.092 | 0.23 | 0.581 | NA | 0.839 | 0.541 | 1 | 0.461 | 0.248 |
| LDL-C_KM-12_ | <0.001 | 0.581 | 1 | 0.296 | 0.458 | 0.063 | 0.227 | 0.296 | NA | 0.109 | 1 | 0.215 | 0.302 |
| LDL-C_KO-12_ | <0.001 | 0.035 | 0.248 | 0.035 | 0.061 | 0.007 | 1 | 0.035 | 0.541 | NA | 0.332 | 0.839 | 0.027 |
| LDL-C_KM-28_ | <0.001 | 0.454 | 0.832 | 0.442 | 0.405 | 0.146 | 0.481 | 0.268 | 1 | 1 | NA | 0.23 | 0.23 |
| LDL-C_KO-28_ | <0.001 | 0.08 | 0.108 | 0.063 | 0.098 | 0.047 | 0.736 | 0.063 | 0.461 | 0.215 | 0.839 | NA | 0.035 |
| LDL-C_KM-180_ | <0.001 | 0.824 | 0.572 | 0.864 | 1 | 1 | 0.031 | 0.864 | 0.248 | 0.302 | 0.027 | 0.23 | NA |

LDL-C: low-density lipoprotein cholesterol; LDL-C_E_: estimated LDL-C; LDL-C_F_: LDL-C calculated using the Friedewald formula; LDL-C_M-N_ (LDL-C_M-10_ and LDL-C_M-180_): LDL-C calculated using the N-cell Martin–Hopkins method described by Martin et al [14]; TG/VLDL-C ratio: ratio of triglycerides to very-low-density lipoprotein cholesterol; LDL-C_KM-N_ (LDL-C_KM-6-TG_, LDL-C_KM-10_, LDL-C_KM-12-TG_, LDL-C_KM-12_, LDL-C_KM-28_, and LDL-C_KM-180_): LDL-C calculated using the N-cell tables with the median TG/VLDL-C ratios derived from our dataset; LDL-C_KO-N_ (LDL-C_KO-6-TG_, LDL-C_KO-10_, LDL-C_KO-12-TG_, LDL-C_KO-12_, and LDL-C_KO-28_): LDL-C calculated using the N-cell tables with the optimal TG/VLDL-C ratios derived from our dataset; NA, not applicable.

*^a^* The values in the table are *p*-values. Statistical significance of differences in overall concordance between two LDL-C estimates was assessed using McNemar’s exact test for correlated proportions.

*^b^* When stratification was based on TG levels alone, rather than combined TG and non–HDL-C levels, the subscript “_TG_” was added, as in LDL-C_KM-N-TG_ or LDL-C_KO-N-TG_.

**S8 Table. Model fit and error metrics for estimated LDL-C (LDL-C_E_) versus directly measured LDL-C (LDL-C_D_): Comparison of Friedewald, Martin–Hopkins, and other estimation methods.**

|  | Model Fit | Error *^a^*, mg/dL | Absolute Error, mg/dL | Squared Error, mg/dL^2^ | Relative Error *^b^* , % |
| --- | --- | --- | --- | --- | --- |
| LDL-C_E_ | *R*-square | Mean (SD) | Mean (SD) | Mean (SD) | Mean (SD) |
| LDL-C_KO-28_ | 0.954 | 0.36 (6.89) | 5.09 (4.66) | 47.64 (236.93) | 0.73 (7.04) |
| Martin–Hopkins [14] | 0.952 | 0.98 (6.94) | 5.11 (4.80) | 49.15 (248.40) | 1.31 (7.21) |
| Sampson [15] | 0.948 | 1.54 (7.30) | 5.59 (4.95) | 55.70 (198.93) | 1.68 (7.31) |
| Rao [16] | 0.946 | 0.87 (7.47) | 5.63 (4.98) | 56.59 (247.93) | 1.06 (7.62) |
| Puavilai [17] | 0.943 | 0.35 (7.62) | 5.75 (5.02) | 58.20 (249.73) | 0.58 (7.74) |
| Chen [18] | 0.951 | –2.12 (7.04) | 5.61 (4.76) | 54.09 (210.74) | –1.24 (7.14) |
| DeLong [19] | 0.947 | 3.24 (7.37) | 6.09 (5.26) | 64.84 (254.78) | 3.32 (7.66) |
| Friedewald [6] | 0.932 | –0.76 (8.36) | 6.26 (5.59) | 70.39 (234.90) | –0.45 (8.57) |
| Vujovic [20] | 0.952 | 5.64 (7.09) | 6.99 (5.76) | 82.07 (283.71) | 5.59 (7.48) |
| McNamara [21] | 0.932 | –2.81 (8.36) | 6.67 (5.77) | 77.75 (236.94) | –2.43 (8.50) |
| Teerakanchana [22] | 0.937 | 4.92 (7.97) | 7.11 (6.09) | 87.65 (257.24) | 5.71 (9.61) |
| Dansethakul [23] | 0.931 | 6.88 (8.39) | 8.92 (6.19) | 117.83 (267.51) | 7.03 (9.29) |
| Hattori [24] | 0.932 | –7.02 (8.24) | 8.50 (6.70) | 117.21 (234.53) | –6.09 (8.09) |
| Saiedullah [25] | 0.952 | 9.92 (6.90) | 10.22 (6.45) | 145.96 (299.36) | 10.26 (9.07) |
| Rasouli [26] | 0.932 | –9.52 (10.17) | 11.37 (8.04) | 193.94 (267.03) | –6.95 (9.47) |
| Anandaraja [27] | 0.763 | 5.60 (15.69) | 12.78 (10.69) | 277.53 (504.85) | 6.97 (18.33) |
| DeCordova [28] | 0.894 | –10.21 (10.72) | 12.51 (7.93) | 219.28 (288.89) | –8.12 (10.08) |
| Ahmadi [29] | 0.398 | 23.26 (44.73) | 33.69 (37.50) | 2541.56 (5798.41) | 21.68 (47.01) |

LDL-C: low-density lipoprotein cholesterol; LDL-C_KO-28_: LDL-C calculated using the 28-cell table (Fig 2) for the optimal TG/VLDL-C ratios derived from our dataset; TG/VLDL-C ratio: ratio of triglycerides to very-low-density lipoprotein cholesterol; SD: standard deviation.

*^a^* Error = LDL-C_E_ – LDL-C_D_.

*^b^* Relative Error = [(LDL-C_E_ – LDL-C_D_) / LDL-C_D_] × 100

LDL-C _Martin–Hopkins_: LDL-C calculated using the original 180-cell Martin–Hopkins equation.

**(A) LDL-C_F_ versus LDL-C_D_	(B) LDL-C_M-180_ versus LDL-C_D_	(C) LDL-C_KO-28_ versus LDL-C_D_**

**Fig 4. Bland–Altman plots comparing estimated LDL-C values with directly measured LDL-C**. Each panel shows the bias (i.e., the difference between LDL-C_E_ and LDL-C_D_) plotted against the mean of LDL-C_D_ and LDL-C_E_. The back dashed line represents the mean bias, while the orange and red dashed lines indicate the upper and lower 95% limit of agreement (mean ± 1.96 SD), respectively. Panels (A), (B), and (C) correspond to LDL-C_F_, LDL-C_M-180_, and LDL-C_KO-28_, respectively. These plots illustrate differences in bias magnitude and dispersion, reflecting the degree of agreement between each LDL-C_E_ and LDL-C_D_. Green dots represent individuals with TG levels of 300–399 mg/dL. **Abbreviations:** LDL-C: low-density lipoprotein cholesterol; TG: triglyceride; LDL-C_E_: estimated LDL-C; LDL-C_D_: LDL-C directly measured using a homogeneous enzyme assay; LDL-C_F_: LDL-C calculated using the Friedewald formula; LDL-C_M-180_: LDL-C calculated using the original 180-cell Martin–Hopkins equation proposed by Martin et al. [14]; LDL-C_KO-28_: LDL-C calculated using the 28-cell table (Fig 2) with the optimal ratios of triglycerides to very-low-density lipoprotein cholesterol (TG/VLDL-C) derived from our dataset; SD: standard deviation.

**Point 3**: Discussion
- One aspect to highlight may be the difference in sample size for the training of the MH equation and the specific equation in the Korean sample. If you can outperform the MH-equation with a smaller subset of training data, there is potential for even better prediction with a larger sample size to train on. Specifically, when talking about using the optimal TG/VLDL-C ratio rather than median, this could be considered a different formula completely, rather than just refitting the MH Equation within your own sample

**Response 3**:

We thank the reviewer for this important and constructive observation. In response, we revised the Discussion section to highlight the substantial difference in the sample size and the conceptual distinction between our method and the original Martin–Hopkins (MH) equation.

The MH equation was developed using a large U.S. dataset of over 900,000 individuals, which enabled fine-grained stratification based on TG and non–HDL-C levels across 180 cells with robust estimation of TG/VLDL-C ratios—an advantage made possible by the ample sample size. In contrast, our study was based on a significantly smaller Korean sample (*n* = 18,322), yet demonstrated that the use of optimal TG/VLDL-C ratios improved LDL-C estimation accuracy compared to using median statistics. For instance, the optimized LDL-C_KO-28_ demonstrated higher concordance than the median-based LDL-C_KM-180_ among individuals with TG levels < 400 mg/dL, even though it used fewer stratification cells. This suggests that substantial performance gains can be achieved even with modestly sized datasets when optimization is tailored to the population and measurement context.

We agree that the implementation of optimal TG/VLDL-C ratios represents a distinct estimation model, rather than merely a recalibration of the Martin–Hopkins method. Accordingly, we have clarified that our model is conceptually grounded in the same estimation framework but restructured through direct optimization using strata-specific concordance with measured LDL-C. This methodological refinement, albeit trained on a smaller dataset, demonstrates strong potential for improved performance and suggests that even better accuracy may be achievable with larger or more diverse cohorts in future applications. In particular, it could enable the incorporation of additional covariates—beyond TG and non–HDL-C—when defining strata for ratio assignment.

We believe this distinction strengthens the translational relevance of our findings and have revised the manuscript accordingly to reflect the independent value and scalability of our approach. This revision is reflected in the Discussion on pages 42–44 as follows:

**Distinct modeling approach and generalizability**

Although the original Martin–Hopkins method was developed using a large U.S. cohort (*n* > 900,000), our study—based on a Korean cohort (*n* = 18,322)—demonstrated that population-specific optimization can yield superior performance. Importantly, our approach does not merely recalibrate the original Martin–Hopkins equation but represents a distinct model that optimizes TG/VLDL-C ratios to enhance concordance with directly measured LDL-C values. For example, despite employing fewer stratification cells, the 28-cell optimized LDL-C_KO-28_ achieved slightly higher concordance (84.0%) than the 180-cell median-based LDL-C_KM-180_ (83.7%) among individuals with TG levels < 400 mg/dL (Table 4). This finding suggests that careful optimization can outperform more granular stratification schemes based on median values, thereby enabling the use of simpler, lower-resolution stratifications.

Such models may offer greater flexibility for incorporating novel, yet potentially performance-enhancing variables. As larger and more diverse datasets become available, the optimized approach can be extended to include additional parameters (e.g., HDL-C, obesity) beyond TG and non–HDL-C, further improving the accuracy of LDL-C estimation.

**Assay-specific and ethnic considerations**

LDL-C measurement techniques may influence the TG/VLDL-C ratios derived for estimation. Our study employed a homogeneous enzymatic assay, while the original Martin–Hopkins method was developed using the VAP technique, which separates lipoprotein fractions through a different process. These methodological differences can substantially impact the estimation of TG/VLDL-C ratios and, in turn, affect the performance and transferability of estimation methods.

This highlights the need for assay-specific recalibration when applying such models in diverse laboratory settings. In addition, ethnic and genetic factors, along with lifestyle and dietary patterns, may contribute to lipid distribution and TG/VLDL-C ratios across populations. While our optimized model improved concordance in a Korean cohort, external validation in diverse ethnic groups and clinical settings is essential to ensure broader applicability and generalizability across populations.

**Practical implementation and scalability**

Many laboratories routinely collect lipid profile data, making local optimization of TG/VLDL-C ratios both feasible and potentially impactful. The appropriate number of stratification cells should be determined by the size and robustness of the available dataset. Our findings demonstrate that even a simplified 6-cell model based solely on TG levels (LDL-C_KO-6-TG_) can substantially improve concordance over LDL-C_F_, particularly among individuals with TG levels of 150–399 mg/dL (Fig 3B). When sufficient data are available, incorporating stratification by non–HDL-C to form a 12-cell model (LDL-C_KO-12_) can enhance performance.

However, increasing the number of strata should be approached with caution. As the number of cells or strata increases, the stability and reliability of the derived TG/VLDL-C ratios may decline, and the resulting performance gains may be only marginal. In settings where local recalibration is not feasible, validated ratio tables from demographically or methodologically similar populations may serve as a practical alternative. Nonetheless, as assay platforms and lipid distributions evolve over time, periodic reassessment and updating of TG/VLDL-C ratios should be integrated into routine quality control practices, particularly when transitioning to new laboratory assays.

**Point 4**: ‘this improvement can achieve without increasing the number of strata,’ in the conclusion has a typo, should be ‘this improvement can be achieved without increasing the number of strata.’

**Response 4**:

We appreciate your careful review and have corrected the phrasing as suggested. The updated sentence appears on page 46 as follows:

. . . Importantly, this improvement can be achieved without increasing the number of strata, offering a practical pathway to refine LDL-C estimation while avoiding excessive stratification.

**Point 5**: A key on what the colors mean in figure 2 would be helpful.

**Response 5**:

Thank you for the helpful suggestion. In response, we have added a color-coded legend to Figure 2 to clarify the ranges of TG/VLDL-C ratios represented by each color band. This revised figure now includes the following color key:

- Blue: TG/VLDL-C ratio < 4.0
- Light Blue: 4.0–4.9
- Green: 5.0–5.9
- Yellow: 6.0–6.9
- Red: ≥ 7.0

This legend has been integrated directly into the figure and is also described in the revised figure caption. For your convenience, the updated figure is included at the end of this response letter. The revised caption appears on pages 18–19 as follows:

**Fig 2. Median and optimal TG/VLDL-C ratios by TG and non–HDL-C strata (28-cell).** The left and right panels show the median and optimal TG/VLDL-C ratios, respectively. Color bands represent the TG/VLDL-C ratio ranges as follows: blue (< 4.0), light blue (4.0–4.9), green (5.0–5.9), yellow (6.0–6.9), and red (≥ 7.0). **Abbreviations:** TG/VLDL-C ratio: the ratio of triglycerides to very-low-density lipoprotein cholesterol; TG: triglyceride; Non–HDL-C: non-high-density lipoprotein cholesterol.

**Fig 2. Median and optimal TG/VLDL-C ratios by TG and non–HDL-C strata (28-cell).** The left and right panels show the median and optimal TG/VLDL-C ratios, respectively. Color bands represent the TG/VLDL-C ratio ranges as follows: blue (< 4.0), light blue (4.0–4.9), green (5.0–5.9), yellow (6.0–6.9), and red (≥ 7.0). **Abbreviations:** TG/VLDL-C ratio: the ratio of triglycerides to very-low-density lipoprotein cholesterol; TG: triglyceride; non–HDL-C: non-high-density lipoprotein cholesterol.
